# Supplementary material for: Pattern and perceived changes in quality of life of Vietnamese medical and nursing students during the COVID-19 pandemic
Source: PLoS One. 2022 Dec 22;17(12):e0279446. doi: 10.1371/journal.pone.0279446 (PMC9778935; doi:10.1371/journal.pone.0279446)
Supplement: S1 File — (DOCX) [file pone.0279446.s001.docx]

**S1 Table. PCS scores categories by reference population average**

| **PCS** | Above population average | Below population average | Total | p-value |
| --- | --- | --- | --- | --- |
| **n (%)** | 530 (33.5) | 1053 (66.5) | 1583 (100.0) |  |
| **Academic majors, n (%)** |  |  |  |  |
| Doctor of Medicine, n (%) | 377 (36.5) | 655 (63.5) | 1032 (100.0) |  |
| Doctor of Preventive Medicine, n (%) | 98 (31.8) | 210 (68.2) | 308 (100.0) |  |
| Nurse, n (%) | 55 (22.6) | 188 (77.4) | 243 (100.0) | **<0.01** |
| **Gender, n (%)** |  |  |  |  |
| Female, n (%) | 294 (29.7) | 697 (70.3) | 991 (100.0) |  |
| Male, n (%) | 236 (39.9) | 356 (60.1) | 592 (100.0) | **<0.01** |
| **Currently on clinical rotation, n (%)** |  |  |  |  |
| No, n (%) | 125 (22.8) | 423 (77.2) | 548 (100.0) |  |
| Yes, n (%) | 405 (39.1) | 630 (60.9) | 1035 (100.0) | **<0.01** |
| **Marital status, n (%)** |  |  |  |  |
| Single, n (%) | 525 (33.4) | 1047 (66.6) | 1572 (100.0) |  |
| Married, n (%) | 5 (45.5) | 6 (54.5) | 11 (100.0) | 0.4 |
| **Affordability of healthcare service, n (%)** |  |  |  |  |
| No Difficulties, n (%) | 302 (36.7) | 521 (63.3) | 823 (100.0) |  |
| Difficulties, n (%) | 228 (30.0) | 532 (70.0) | 760 (100.0) | **<0.01** |
| **BMI categories, n (%)** |  |  |  |  |
| Underweight, n (%) | 90 (28.0) | 232 (72.0) | 322 (100.0) |  |
| Normal, n (%) | 356 (34.7) | 671 (65.3) | 1027 (100.0) |  |
| Overweight, n (%) | 77 (36.8) | 132 (63.2) | 209 (100.0) |  |
| Obese, n (%) | 7 (28.0) | 18 (72.0) | 25 (100.0) | 0.09 |
| **Having chronic disease, n (%)** |  |  |  |  |
| No, n (%) | 495 (33.7) | 973 (66.3) | 1468 (100.0) |  |
| Yes, n (%) | 35 (30.4) | 80 (69.6) | 115 (100.0) | 0.47 |
| **Symptoms of Covid-19, n (%)** |  |  |  |  |
| Asymptomic, n (%) | 471 (36.7) | 811 (63.3) | 1282 (100.0) |  |
| Non-typical symptoms, n (%) | 14 (14.1) | 85 (85.9) | 99 (100.0) |  |
| Typical symptoms, n (%) | 45 (22.3) | 157 (77.7) | 202 (100.0) | **<0.01** |
| **FCV-19S scores categories, n(%)** |  |  |  |  |
| Low, n (%) | 415 (37.3) | 697 (62.7) | 1112 (100.0) |  |
| High, n (%) | 115 (24.4) | 356 (75.6) | 471 (100.0) | **<0.01** |
| Statistical comparison using: | | | | |
| Chi-square test for categorical variable - display as n(%) (1); | | | | |
| T test for continuous-normally distributed variable - display as mean(sd) (2); | | | | |
| Wilcoxon rank-sum test for continuous-skewed variable - display as median(iqr) (3); | | | | |
| Bold p-value indicated statistical significance (p<0.05). | | | | |
| N/A: Not applicable | | | | |

**S2 Table. MCS scores categories by reference population average**

| **MCS** | Above population average | Below population average | Total | P-value |
| --- | --- | --- | --- | --- |
| **n (%)** | 28 (1.8) | 1555 (98.2) | 1583 (100.0) |  |
| **Academic majors, n (%)** |  |  |  |  |
| Doctor of Medicine, n (%) | 22 (2.1) | 1010 (97.9) | 1032 (100.0) |  |
| Doctor of Preventive Medicine, n (%) | 4 (1.3) | 304 (98.7) | 308 (100.0) |  |
| Nurse, n (%) | 2 (0.8) | 241 (99.2) | 243 (100.0) | 0.3 |
| **Gender, n (%)** |  |  |  |  |
| Female, n (%) | 15 (1.5) | 976 (98.5) | 991 (100.0) |  |
| Male, n (%) | 13 (2.2) | 579 (97.8) | 592 (100.0) | 0.32 |
| **Currently on clinical rotation, n (%)** |  |  |  |  |
| No, n (%) | 9 (1.6) | 539 (98.4) | 548 (100.0) |  |
| Yes, n (%) | 19 (1.8) | 1016 (98.2) | 1035 (100.0) | 0.78 |
| **Marital status, n (%)** |  |  |  |  |
| Single, n (%) | 28 (1.8) | 1544 (98.2) | 1572 (100.0) |  |
| Married, n (%) | 0 (0.0) | 11 (100.0) | 11 (100.0) | 0.66 |
| **Affordability of healthcare service, n (%)** |  |  |  |  |
| No Difficulties, n (%) | 16 (1.9) | 807 (98.1) | 823 (100.0) |  |
| Difficulties, n (%) | 12 (1.6) | 748 (98.4) | 760 (100.0) | 0.58 |
| **BMI categories, n (%)** |  |  |  |  |
| Underweight, n (%) | 2 (0.6) | 320 (99.4) | 322 (100.0) |  |
| Normal, n (%) | 21 (2.0) | 1006 (98.0) | 1027 (100.0) |  |
| Overweight, n (%) | 5 (2.4) | 204 (97.6) | 209 (100.0) |  |
| Obese, n (%) | 0 (0.0) | 25 (100.0) | 25 (100.0) | 0.28 |
| **Having chronic disease, n (%)** |  |  |  |  |
| No, n (%) | 27 (1.8) | 1441 (98.2) | 1468 (100.0) |  |
| Yes, n (%) | 1 (0.9) | 114 (99.1) | 115 (100.0) | 0.45 |
| **Symptoms of Covid-19, n (%)** |  |  |  |  |
| No symptoms, n (%) | 25 (2.0) | 1257 (98.0) | 1282 (100.0) |  |
| Atypical symptoms, n (%) | 0 (0.0) | 99 (100.0) | 99 (100.0) |  |
| Typical symptoms, n (%) | 3 (1.5) | 199 (98.5) | 202 (100.0) | 0.35 |
| **FCV-19S scores categories, n(%)** |  |  |  |  |
| Low, n (%) | 25 (2.2) | 1087 (97.8) | 1112 (100.0) |  |
| High, n (%) | 3 (0.6) | 468 (99.4) | 471 (100.0) | **0.03** |
| Statistical comparison using: | | | | |
| Chi-square test for categorical variable - display as n(%) (1); | | | | |
| T test for continuous-normally distributed variable - display as mean(sd) (2); | | | | |
| Wilcoxon rank-sum test for continuous-skewed variable - display as median(iqr) (3); | | | | |
| Bold p-value indicated statistical significance (p<0.05). | | | | |
| N/A: Not applicable | | | | |

**S3 Fig. Residuals versus fitted values plot of PCS scores linear regression model**


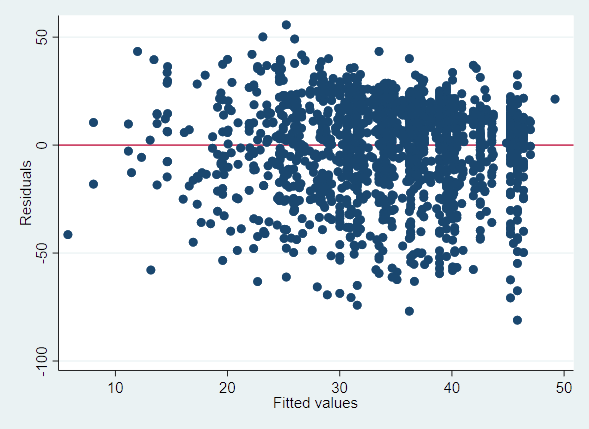


**S4 Fig. Residuals versus fitted values plot of MCS scores linear regression model**
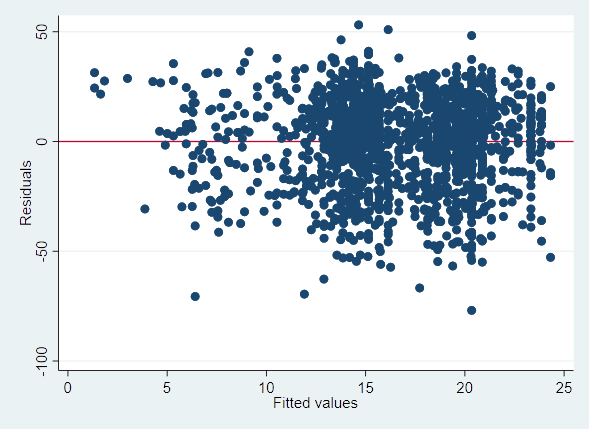


**S5 Table. Quantile regression analysis of PCS**

|  | **Physical Composite Score** | | |  |
| --- | --- | --- | --- | --- |
|  | **Coef.** | **p-value** | **95%** | **CI** |
| **Academic Major** |  |  |  |  |
| General Medicine | 0.00 | (base) |  |  |
| Preventive Medicine | -3.00 | 0.121 | -6.8 | 0.79 |
| Nursing | -6.41 | 0.004 | -11 | -2.1 |
| **Gender** |  |  |  |  |
| Female | 0.00 | (base) |  |  |
| Male | 2.61 | 0.121 | -0.69 | 5.9 |
| **Currently on Clinical Rotation** |  |  |  |  |
| No | 0.00 | (base) |  |  |
| Yes | 10.70 | 0 | 7.6 | 14 |
| **Marital Status** |  |  |  |  |
| Single | 0.00 | (base) |  |  |
| Married | 2.27 | 0.794 | -15 | 19 |
| **Affordabilities of Healthcare Services** |  |  |  |  |
| No Difficulties | 0.00 | (base) |  |  |
| Difficulties | -6.29 | 0 | -9.2 | -3.4 |
| **BMI Category** |  |  |  |  |
| Underweight | 0.00 | (base) |  |  |
| Normal | 2.65 | 0.154 | -1 | 6.3 |
| Overweight | 3.79 | 0.161 | -1.5 | 9.1 |
| Obese | -2.53 | 0.676 | -14 | 9.3 |
| **Having Chronic Disease** |  |  |  |  |
| No | 0.00 | (base) |  |  |
| Yes | -2.05 | 0.465 | -7.5 | 3.4 |
| **Symptoms of Covid-19** |  |  |  |  |
| No symptoms | 0.00 | (base) |  |  |
| Typical Symptoms | -5.96 | 0.007 | -10 | -1.6 |
| Atypical Symptoms | -14.26 | 0 | -20 | -8.3 |
| **Fear of Covid Scale score categories** |  |  |  |  |
| Low | 0.00 | (base) |  |  |
| High | -7.12 | 0 | -10 | -4 |

**S6 Table. Quantile regression analysis of MCS**

|  | **Mental Composite Score** | | |  |
| --- | --- | --- | --- | --- |
|  | **Coef.** | **p-value** | **95%** | **CI** |
| **Academic Major** |  |  |  |  |
| General Medicine | 0 | (base) |  |  |
| Preventive Medicine | 2.96 | 0.064 | -0.17 | 6.1 |
| Nursing | 1.02 | 0.576 | -2.6 | 4.6 |
| **Gender** |  |  |  |  |
| Female | 0 | (base) |  |  |
| Male | 1.69 | 0.225 | -1 | 4.4 |
| **Currently on Clinical Rotation** |  |  |  |  |
| No | 0 | (base) |  |  |
| Yes | 3.239 | 0.012 | 0.71 | 5.8 |
| **Marital Status** |  |  |  |  |
| Single | 0 | (base) |  |  |
| Married | -9.881 | 0.169 | -24 | 4.2 |
| **Affordabilities of Healthcare Services** |  |  |  |  |
| No Difficulties | 0 | (base) |  |  |
| Difficulties | -6.257 | 0 | -8.6 | -3.9 |
| **BMI Category** |  |  |  |  |
| Underweight | 0 | (base) |  |  |
| Normal | 0.2727 | 0.859 | -2.7 | 3.3 |
| Overweight | 0.8481 | 0.704 | -3.5 | 5.2 |
| Obese | -3.145 | 0.529 | -13 | 6.7 |
| **Having Chronic Disease** |  |  |  |  |
| No | 0 | (base) |  |  |
| Yes | -4.15 | 0.073 | -8.7 | 0.4 |
| **Symptoms of Covid-19** |  |  |  |  |
| No symptoms | 0 | (base) |  |  |
| Typical Symptoms | -2.829 | 0.121 | -6.4 | 0.74 |
| Atypical Symptoms | -7.695 | 0.002 | -13 | -2.8 |
| **Fear of Covid Scale score categories** |  |  |  |  |
| Low | 0 | (base) |  |  |
| High | -1.599 | 0.228 | -4.2 | 1 |

**S7 Table. Changes in quality of life before and after the pandemic**

| **Columns by: Changes in Quality of Life** | **Not Worse** | **Worse** | **Total** | **P-value** |
| --- | --- | --- | --- | --- |
| n (%) | 1427 (90.1) | 156 (9.9) | 1583 (100.0) |  |
| **Age, mean (sd)** | 21.55 (1.94) | 22.47 (2.30) | 21.64 (2.00) | **<0.01** |
| **Academic majors, n (%)** |  |  |  |  |
| General Medicine, n (%) | 932 (90.3) | 100 (9.7) | 1032 (100.0) |  |
| Preventive Medicine, n (%) | 273 (88.6) | 35 (11.4) | 308 (100.0) |  |
| Nursing, n (%) | 222 (91.4) | 21 (8.6) | 243 (100.0) | 0.54 |
| **Gender, n (%)** |  |  |  |  |
| Female, n (%) | 900 (90.8) | 91 (9.2) | 991 (100.0) |  |
| Male, n (%) | 527 (89.0) | 65 (11.0) | 592 (100.0) | 0.25 |
| **Academic years, n (%)** |  |  |  |  |
| First year, n (%) | 254 (90.1) | 28 (9.9) | 282 (100.0) |  |
| Second year, n (%) | 329 (95.4) | 16 (4.6) | 345 (100.0) |  |
| Third year, n (%) | 211 (93.4) | 15 (6.6) | 226 (100.0) |  |
| Fourth year, n (%) | 176 (94.1) | 11 (5.9) | 187 (100.0) |  |
| Fifth year, n (%) | 180 (91.8) | 16 (8.2) | 196 (100.0) |  |
| Sixth year, n (%) | 277 (79.8) | 70 (20.2) | 347 (100.0) | **<0.01** |
| **Currently on clinical rotation, n (%)** |  |  |  |  |
| No, n (%) | 510 (93.1) | 38 (6.9) | 548 (100.0) |  |
| Yes, n (%) | 917 (88.6) | 118 (11.4) | 1035 (100.0) | **<0.01** |
| **Marital status, n (%)** |  |  |  |  |
| Single, n (%) | 1418 (90.2) | 154 (9.8) | 1572 (100.0) |  |
| Married, n (%) | 9 (81.8) | 2 (18.2) | 11 (100.0) | 0.35 |
| **Affordability of healthcare services, n (%)** |  |  |  |  |
| No Difficulties, n (%) | 756 (91.9) | 67 (8.1) | 823 (100.0) |  |
| Difficulties, n (%) | 671 (88.3) | 89 (11.7) | 760 (100.0) | **0.02** |
| **BMI categories, n (%)** |  |  |  |  |
| Underweight, n (%) | 294 (91.3) | 28 (8.7) | 322 (100.0) |  |
| Normal, n (%) | 926 (90.2) | 101 (9.8) | 1027 (100.0) |  |
| Overweight, n (%) | 189 (90.4) | 20 (9.6) | 209 (100.0) |  |
| Obese, n (%) | 18 (72.0) | 7 (28.0) | 25 (100.0) | **0.02** |
| **Having chronic disease, n (%)** |  |  |  |  |
| No, n (%) | 1336 (91.0) | 132 (9.0) | 1468 (100.0) |  |
| Yes, n (%) | 91 (79.1) | 24 (20.9) | 115 (100.0) | **<0.01** |
| **Symptoms of Covid-19, n (%)** |  |  |  |  |
| No symptoms, n (%) | 1177 (91.8) | 105 (8.2) | 1282 (100.0) |  |
| Typical Symptoms, n (%) | 170 (84.2) | 32 (15.8) | 202 (100.0) |  |
| Atypical Symptoms, n (%) | 80 (80.8) | 19 (19.2) | 99 (100.0) | **<0.01** |
| **FCV-19S score categories, n (%)** |  |  |  |  |
| Low, n (%) | 993 (89.3) | 119 (10.7) | 1112 (100.0) |  |
| High, n (%) | 434 (92.1) | 37 (7.9) | 471 (100.0) | 0.08 |
| **FCV-19S score, median (iqi)** | 16.00 (13.00; 21.00) | 16.00 (13.00; 20.00) | 16.00 (13.00; 21.00) | 0.68 |
| Statistical comparison using | | | | |
| Chi-square test for categorical variable - display as n(%); | | | | |
| T test for continuous-normally distributed variable - display as mean(sd); | | | | |
| Wilcoxon rank-sum test for continuous-skewed variable - display as median(iqi); | | | | |
| The bold p-value indicated statistical significance (p<0.05). | | | | |

**S8 Table.** **SF-36 PCS and MCS scores with z-score derived from USA population averages**

|  | **PCS** | | | **MCS** | | |
| --- | --- | --- | --- | --- | --- | --- |
|  | Median | P25 | P75 | Median | P25 | P75 |
| **Academic majors** |  |  |  |  |  |  |
| General Medicine | 54 | 48.4 | 57.4 | 43.5 | 37.8 | 48.7 |
| Preventive Medicine | 53.4 | 47.7 | 57 | 44.3 | 39.3 | 50.2 |
| Nursing | 50.9 | 45.5 | 55.3 | 42.6 | 36.6 | 47.5 |
|  |  |  |  |  |  |  |
| **Gender** |  |  |  |  |  |  |
| Female | 53 | 47.7 | 56.7 | 43.4 | 37.8 | 48.3 |
| Male | 54.6 | 47.4 | 57.5 | 43.8 | 37.8 | 49.2 |
|  |  |  |  |  |  |  |
| **Academic years** |  |  |  |  |  |  |
| First year | 50.3 | 44.9 | 54.6 | 42.9 | 36.6 | 48.6 |
| Second year | 52.8 | 47 | 56.7 | 43.2 | 38.2 | 48.6 |
| Third year | 53 | 47.8 | 56.9 | 43.3 | 37.1 | 46.7 |
| Fourth year | 55.3 | 50.8 | 57.5 | 43.4 | 38.4 | 49.8 |
| Fifth year | 55.4 | 50.4 | 58.1 | 45.2 | 39.9 | 49.2 |
| Sixth year | 54.4 | 48.6 | 57.6 | 44.1 | 37.2 | 50.2 |
|  |  |  |  |  |  |  |
| **Academic Year** |  |  |  |  |  |  |
| First-year | 50.3 | 44.9 | 54.6 | 42.9 | 36.6 | 48.6 |
| Middle-year | 54 | 48.3 | 57.3 | 43.6 | 38.3 | 48.4 |
| Final-Year | 54.5 | 49 | 57.6 | 43.9 | 37.3 | 50 |
|  |  |  |  |  |  |  |
| **Currently on clinical rotation** | |  |  |  |  |  |
| No | 51.4 | 45.7 | 55.9 | 42.9 | 37.4 | 48.6 |
| Yes | 54.5 | 48.7 | 57.5 | 43.9 | 38 | 48.8 |
|  |  |  |  |  |  |  |
| Marital status |  |  |  |  |  |  |
| Single | 53.4 | 47.7 | 57.1 | 43.6 | 37.8 | 48.7 |
| Married | 55.7 | 46 | 57.9 | 41.3 | 37.5 | 51.9 |
|  |  |  |  |  |  |  |
| **Affordability of healthcare services** | | |  |  |  |  |
| No Difficulties | 54.4 | 49.2 | 57.4 | 45 | 39.5 | 50.1 |
| Difficulties | 52.2 | 46.3 | 56.7 | 42.1 | 36.1 | 47.3 |
|  |  |  |  |  |  |  |
| **BMI categories** |  |  |  |  |  |  |
| Underweight | 52.2 | 46.6 | 56.2 | 42.8 | 37 | 47.5 |
| Normal | 53.7 | 47.8 | 57.4 | 43.7 | 38.1 | 48.8 |
| Overweight | 54.5 | 49 | 57.2 | 44.6 | 37.6 | 49 |
| Obese | 52.5 | 47.4 | 55.7 | 44.4 | 38.1 | 50.6 |
|  |  |  |  |  |  |  |
| **Symptoms of Covid-19** |  |  |  |  |  |  |
| No symptoms | 54 | 48.3 | 57.4 | 43.8 | 38.6 | 48.9 |
| Typical Symptoms | 52 | 46.2 | 55.6 | 43 | 36.1 | 47.9 |
| Atypical Symptoms | 48.4 | 43.7 | 54 | 40.3 | 33.2 | 44.9 |
|  |  |  |  |  |  |  |
| **Having chronic disease** |  |  |  |  |  |  |
| No | 53.4 | 47.7 | 57.1 | 43.7 | 38 | 48.8 |
| Yes | 53.1 | 47.5 | 56.5 | 42.8 | 34.7 | 46.6 |
|  |  |  |  |  |  |  |
| **FCV-19S score categories** | |  |  |  |  |  |
| Low | 54.2 | 48.7 | 57.5 | 44.3 | 38.1 | 49.3 |
| High | 51.2 | 45.5 | 56 | 42.5 | 37.5 | 46.5 |
|  |  |  |  |  |  |  |
| **Changes in Quality of Life** | |  |  |  |  |  |
| Not Worse | 53.7 | 48.3 | 57.2 | 44.1 | 39.2 | 49 |
| Worse | 48.8 | 42.6 | 55.6 | 36 | 30.9 | 43.3 |
|  |  |  |  |  |  |  |
| Total | 53.4 | 47.7 | 57.1 | 43.5 | 37.8 | 48.7 |
