# Supplementary material for: Pattern and perceived changes in quality of life of Vietnamese medical and nursing students during the COVID-19 pandemic
Source: PLoS One. 2022 Dec 22;17(12):e0279446. doi: 10.1371/journal.pone.0279446 (PMC9778935; doi:10.1371/journal.pone.0279446)
Supplement: Attachment — Submitted filename: Rebu letter.docx [file pone.0279446.s002.docx]

Dear Editors,

Sincerest thanks for the previous editor’s and reviewers’ comments on our manuscript. We hope that a revised version of the manuscript will still be considered by PLOS One.

We have modified the paper in response to the extensive and insightful reviewers’ comments. Furthermore, we have rewritten sections of the manuscript and hope that this complies with the referee’s remarks.

We will respond to the comments point by point.

Our answers to reviews’ question were highlighted in yellow

PONE-D-22-11803
Pattern and perceived changes in quality of life of Vietnamese medical and nursing students during the COVID-19 pandemic
PLOS ONE

Dear Dr. Pham,

Thank you very much for submitting your manuscript for review by PLOS ONE. After careful consideration, we have decided that your manuscript does not meet our criteria and is therefore not suitable for publication in PLOS ONE.

After internal review of your manuscript, we had several concerns with the analyses, conclusions and quality of the scientific reporting. Please note that as per our publication criteria, PLOS ONE requires that all experiments, statistics and other analyses are performed to a high technical standard, described in sufficient detail and adhere to appropriate reporting guidelines and community standards. Conclusions must be presented in an appropriate fashion and be supported by the data (please see <http://journals.plos.org/plosone/s/criteria-for-publication>). After careful consideration, we feel that the current manuscript does not meet these requirements.

For example you have not provided us with sufficient information regarding the steps taken to fully validate the additional questionnaire questions used for data collection in the study.

Thank you for your comments. We added the following information to our method section:

“The questionnaire included questions regarding *demographic information*, *academic information* and *health information*, *the Fear of Covid 19 Scale (FCV-19S)*, and the *Quality of life SF-36 version 2.0 (SF-36)*, and *a question asking about the change in the general quality of life before and during the pandemic*.”

“*Fear of Covid-19* was assessed using the FCV-19S questionnaire. The FCV-19S questionnaire was translated into Vietnamese in a previous study and showed good item-scale convergent validity (mean of Rho = 0.77), discriminant validity and construct validity and high internal consistency (Cronbach’s alpha = 0.90) with the Vietnamese translation [32,33].”

“The Vietnamese version of the SF-36 was validated and was shown to provide a valid assessment of self-reported health status among the Vietnamese population, with all but two of the 36 items displayed good discriminant validity and all eight scale had good discriminant validity displayed the internal consistency of the 8 scales: PF (0.82), RP (0.86), BP (0.58), GH (0.66), VT(0.56), SF (0.67), RE (0.70), MH (0.55) [36].”

As you can see, all of the main questionnaires were validated in previous studies among Vietnamese population. Furthermore, additional information in the survey were just general demographic information, academic information, and health information, such as age, gender, height, weight. Finally, the main aim of this study is not validating these questionnaires (as they were already validated in previous studies among the same population)

In addition, you have not provided sufficient details on the statistical calculations employed to determine the sample size required to power the study.

Thank you for your comments. We added the following information to our method section:

**Sample size and data collection**

The required sample size was estimated by using the formula for estimating sample mean [37], as follows:

$n=\frac{Z_{1-\frac{\alpha}{2}}^{2}{*\sigma}^{2}}{d^{2}}=\frac{{1.96}^{2}*{12.5}^{2}}{1^{2}}=600.25$

With, n being the required sample size, Z being the standard error associated with the chosen level of confidence 5%, σ being the standard deviation of the general quality of life score taken from the population used to validate the Vietnamese version of the SF-36 [36].

For these reasons, we are rejecting your manuscript. I am sorry that we cannot be more positive on this occasion, but hope that you understand the reasons for this decision.

Kind regards,

Marianne Clemence
Staff Editor
PLOS ONE

Additional Editor Comments (if provided):


[Note: HTML markup is below. Please do not edit.]

Reviewers' comments:


[NOTE: If reviewer comments were submitted as an attachment file, they will be attached to this email and accessible via the submission site. Please log into your account, locate the manuscript record, and check for the action link "View Attachments". If this link does not appear, there are no attachment files.]

- - - - -
For journal use only: PONEDEC3
