# Supplementary material for: Pattern and perceived changes in quality of life of Vietnamese medical and nursing students during the COVID-19 pandemic
Source: PLoS One. 2022 Dec 22;17(12):e0279446. doi: 10.1371/journal.pone.0279446 (PMC9778935; doi:10.1371/journal.pone.0279446)
Supplement: Attachment — Submitted filename: 08 June SZ PLOS ONE 2022.docx [file pone.0279446.s003.docx]

PLOS ONE

Pattern and perceived changes in quality of life of Vietnamese medical and nursing students during the COVID-19 pandemic

--Manuscript Draft--

| Manuscript Number: | PONE-D-22-14424 |
| --- | --- |
| Article Type: | Research Article |
| Full Title: | Pattern and perceived changes in quality of life of Vietnamese medical and nursing students during the COVID-19 pandemic |
| Short Title: | Quality of life of Vietnamese medical and nursing student during COVID-19 |
| Corresponding Author: | Tung Thanh Pham, MD, MPH  Hanoi Medical University  Hanoi, Hanoi VIET NAM |
| Keywords: | quality of life; SF36; medical and nursing student; COVID-19 |
| Abstract: | Background  The COVID 19 pandemic and governments’ response lead to dramatical change in quality of life worldwide. However, the extent of this change in Vietnamese medical students has not been documented.  Objectives  The study aims to describe the quality of life and changes in quality of life of medical and nursing students during the COVID-19 pandemic and examine the association of quality of life and changes in quality of life with fear of covid and other socio-economic and demographic factors.  Methods  The study was a cross-sectional study on all students of Hanoi Medical University from  3 majors: General Medicine, Preventive Medcine, Nursing; from 7th to 29th of April 2020; using an online questionnaire that included demographic and academic information, the Vietnamese version of the SF-36 Quality of Life questionnaire and the Fear of COVID-19 Scale (FCV-19S). Linear and modified Poisson regression was used to examine the association between quality of life, changes in quality of life and other factors.  Results  The number of participants was 1583 (response rate 43%). Among 8 dimensions of the SF-36 (ranged 0-100), Vitality had the lowest score with a median score of 46. The median physical composite score (PCS) of the sample was 40.6 (IQR:20.8–53.2),  33.5% of the sample had an above-population average PCS score. The median mental composite score (MCS) of the sample was 20.3 (IQR:3.8–31.7), and 98.2% had an MCS score below average. 9.9% (95%CI:8.5%–11.4%) of the population reported a significant negative change in the quality of life. Fear of COVID-19 was not associated with significant changes in quality of life, nor MCS while having some association with PCS (Coef:-5.39;95%CI:-3to-7.8). Perceived reduction in quality of life was also associated with: being on clinical rotation COVID-19 (PR:1.5;95%CI:1.05–2.2), difficulties affording health services (PR:1.4;95%CI:1.02–1.95), obesity  (PR:2.38;95%CI:1.08–5.25) and chronic disease (PR:1.92;95%CI:1.23–3), typical symptoms (PR:1.85; 95%CI:1.23–2.78) and atypical symptoms of COVID-19 (PR:2.32;95%CI:1.41–3.81).  Conclusion  The majority of medical and nursing students had below average quality of life, with lower vitality and mental composite health score in the settings of COVID-19. Perceived decrease in quality of life was associated with clinical rotation, difficulties affording healthcare services and was not associated with Fear of COVID-19. |
| Order of Authors: | Le Dai Minh |
|  | Hoang Huy Phan |
|  | Duong Ngoc Le Mai |
|  | Nguyen Tien Dat |
|  | Ngo Minh Tri |

|  | Nguyen Viet Ha |
| --- | --- |
|  | Nguyen Huu Tu |
|  | Kirsty Foster |
|  | Kim Bao Giang |
|  | Tung Thanh Pham, MD, MPH |
| Additional Information: |  |
| Question | Response |
| Financial Disclosure  Enter a financial disclosure statement that describes the sources of funding for the work included in this submission. Review the [submission guidelines](http://journals.plos.org/plosone/s/submission-guidelines#loc-financial-disclosure-statement) for detailed requirements. View published research articles from [PLOS ONE](http://journals.plos.org/plosone/) for specific examples.  This statement is required for submission and will appear in the published article if the submission is accepted. Please make sure it is accurate. | The author(s) received no specific funding for this work. |
| Unfunded studies Enter: The author(s) received no specific funding for this work. Funded studies Enter a statement with the following details: - Initials of the authors who received each award - Grant numbers awarded to each author - The full name of each funder - URL of each funder website - Did the sponsors or funders play any role in the study design, data collection and analysis, decision to publish, or preparation of the manuscript? - NO - Include this sentence at the end of your statement: The funders had no role in study design, data collection and analysis, decision to publish, or preparation of the manuscript. - YES - Specify the role(s) played. |  |
| * typeset |  |
| Competing Interests  Use the instructions below to enter a competing interest statement for this submission. On behalf of all authors, | The authors have declared that no competing interests exist. |

| disclose any [competing interests](http://journals.plos.org/plosone/s/competing-interests) that could be perceived to bias this work—acknowledging all financial support and any other relevant financial or nonfinancial competing interests.  This statement is required for submission and will appear in the published article if the submission is accepted. Please make sure it is accurate and that any funding sources listed in your Funding Information later in the submission form are also declared in your Financial Disclosure statement.  View published research articles from [PLOS ONE](http://journals.plos.org/plosone/) for specific examples. |  |
| --- | --- |
| NO authors have competing interests Enter: The authors have declared that no competing interests exist. Authors with competing interests Enter competing interest details beginning with this statement: I have read the journal's policy and the authors of this manuscript have the following competing interests: [insert competing interests here] |  |
| * typeset |  |
| Ethics Statement  Enter an ethics statement for this submission. This statement is required if the study involved:   - Human participants - Human specimens or tissue - Vertebrate animals or cephalopods - Vertebrate embryos or tissues - Field research   Write "N/A" if the submission does not require an ethics statement. | Our survey was approved by the Institutional Review Board of Hanoi Universities of Public Health and Hanoi Medical University (IRB No. 133/2020/YTCC-HD3). The online questionnaire provided participants with comprehensive information about the study. All participants gave informed consent by clicking “I agree to participate” box on the informed consent page. This consent procedure was approved by the IRB. Moreover, the participants could stop participating and close the Google Form at any time during the survey |

General guidance is provided below.

Consult the

[submission guideline](http://journals.plos.org/plosone/s/submission-guidelines#loc-guidelines-for-specific-study-types)

[s](http://journals.plos.org/plosone/s/submission-guidelines#loc-guidelines-for-specific-study-types)

for

detailed instructions.

Make sure that all

information entered here is included in the

Methods section of the manuscript.

Format for specific study types

Human Subject Research (involving human

participants and/or tissue)

Give the name of the institutional review

board or ethics committee that approved the

study

•

Include the approval number and/or a

statement indicating approval of this

research

•

Indicate the form of consent obtained

(

written/oral) or the reason that consent was

not obtained (e.g. the data were analyzed

anonymously)

•

Animal Research (involving vertebrate

animals, embryos or tissues)

Provide the name of the Institutional Animal

Care and Use Committee (IACUC) or other

relevant ethics board that reviewed the

study protocol, and indicate whether they

approved this research or granted a formal

waiver of ethical approval

•

Include an approval number if one was

obtained

•

If the study involved

non-human primates

,

add

additional details

about animal welfare

and steps taken to ameliorate suffering

•

If anesthesia, euthanasia, or any kind of

animal sacrifice is part of the study, include

briefly which substances and/or methods

were applied

•

Field Research

Include the following details if this study

involves the collection of plant, animal, or

other materials from a natural setting:

Field permit number

•

Name of the institution or relevant body that

granted permission

•

Data Availability

Authors are required to make all data

No - some restrictions will apply

| underlying the findings described fully available, without restriction, and from the time of publication. PLOS allows rare exceptions to address legal and ethical concerns. See the [PLOS Data Policy](http://journals.plos.org/plosone/s/data-availability) and [FAQ](http://journals.plos.org/plosone/s/data-availability#loc-faqs-for-data-policy) for detailed information.  A Data Availability Statement describing where the data can be found is required at submission. Your answers to this question constitute the Data Availability Statement and will be published in the article, if accepted. |  |
| --- | --- |
| Important: Stating ‘data available on request from the author’ is not sufficient. If your data are only available upon request, select ‘No’ for the first question and explain your exceptional situation in the text box. |  |
| Do the authors confirm that all data underlying the findings described in their manuscript are fully available without restriction? |  |
| Describe where the data may be found in full sentences. If you are copying our sample text, replace any instances of XXX with the appropriate details.   - If the data are held or will be held in a public repository, include URLs, accession numbers or DOIs. If this information will only be available after acceptance, indicate this by ticking the box below. For example: All XXX files   are available from the XXX database (accession number(s) XXX, XXX.).   - If the data are all contained within the manuscript and/or Supporting Information files, enter the following: All relevant data are within the manuscript and its Supporting Information files. - If neither of these applies but you are able to provide details of access elsewhere, with or without limitations, please do so. For example:   Data cannot be shared publicly because of [XXX]. Data are available from the | According to our application to the Institutional Review Board of Hanoi University of Public Health, the data cannot be shared publicly because of ethical restrictions to protect the confidentiality of the participants. A de-identified dataset is available for researchers who meet the criteria for access to confidential data. Requests for data should be submitted to Institutional Review Board of Hanoi University of Public Health  (irb@huph.edu.vn) and the corresponding author, Dr. Pham Thanh Tung (phamthanhtung@hmu.edu.vn) |

| XXX Institutional Data Access / Ethics Committee (contact via XXX) for researchers who meet the criteria for access to confidential data.  The data underlying the results presented in the study are available from (include the name of the third party and contact information or URL).  • This text is appropriate if the data are owned by a third party and authors do not have permission to share the data.  * typeset |  |
| --- | --- |
| Additional data availability information: | Tick here if your circumstances are not covered by the questions above and you need the journal’s help to make your data available. |

Manuscript clean verision [Click here to access/download;Manuscript;QoL_Vietnamese students_COVID-19_18052022_CLEAN.docx](https://www.editorialmanager.com/pone/download.aspx?id=31182389&guid=0f109066-cc91-4ffc-8908-f410ef102f43&scheme=1) **Pattern and perceived changes in quality of life of Vietnamese medical and nursing students during the COVID-19 pandemic**

**Short title: Quality of life of Vietnamese medical and nursing student during COVID19**

**Le Dai Minh^1^, Hoang Huy Phan^1^, Duong Ngoc Le Mai^1^, Nguyen Tien Dat^1^, Ngo Minh**

# Tri^1^, Nguyen Viet Ha^1^, Nguyen Huu Tu^2^, Kirsty Foster^3^, Kim Bao Giang^4^, Pham Thanh Tung5*

1 Doctor of Medicine Program, Hanoi Medical University, Hanoi, Vietnam
2 Department of Anesthesia, Hanoi Medical University, Hanoi, Vietnam
3 Academy for Medical Education, Faculty of Medicine, University of Queensland, Brisbane, Australia
4 School of Preventive Medicine and Public Health, Hanoi Medical University, Hanoi, Vietnam
5 Department of Physiology, Hanoi Medical University, Hanoi, Vietnam

**^*^** Corresponding author

Email: phamthanhtung@hmu.edu.vn

1. Le Dai Minh

Doctor of Medicine Program, Hanoi Medical University, Hanoi, Vietnam

Email: ledaiminh7499@gmail.com

Orcid ID: 0000-0002-0684-6408

2. Hoang Huy Phan

Doctor of Medicine Program, Hanoi Medical University, Hanoi, Vietnam

Email: hoanghuyphan.chl.toan1518@gmail.com

3. Duong Ngoc Le Mai

Doctor of Medicine Program, Hanoi Medical University, Hanoi, Vietnam

Email: duongngoclemai@gmail.com

OCRID: https://orcid.org/0000-0002-5926-0443

4. Nguyen Tien Dat

Doctor of Medicine Program, Hanoi Medical University, Hanoi, Vietnam Email: dat06111999@gmail.com <https://orcid.org/0000-0002-6083-7043>

5. Ngo Minh Tri

Doctor of Medicine Program, Hanoi Medical University, Hanoi, Vietnam

Email: tringominh99@gmail.com

6. Nguyen Viet Ha

Doctor of Medicine Program, Hanoi Medical University, Hanoi, Vietnam

Email: vhnguyen1812@gmail.com

Orcid ID: [https://orcid.org/0000-0002-6810-4864](https://orcid.org/0000-0002-6810-4864?fbclid=IwAR2VUMosd8GoMINaITz1gBuUA2svhxQ6AWSkhvGDC2cvvH1_NAD3AivfeHY)

7. Nguyen Huu Tu

Department of Anesthesia, Hanoi Medical University, Hanoi, Vietnam

Email: nguyenhuutu@hmu.edu.vn

8. Kirsty Foster

Academy for Medical Education, Faculty of Medicine, University of Queensland, Brisbane,

Australia

Email: kirsty.foster@uq.edu.au

9. Kim Bao Giang

School of Preventive Medicine and Public Health, Hanoi Medical University, Hanoi, Vietnam

Email: kimbaogiang@hmu.edu.vn

10. Pham Thanh Tung

Department of Physiology, Hanoi Medical University, Hanoi, Vietnam

61	Email: phamthanhtung@hmu.edu.vn

62

63

# Abstract

## Background

The COVID 19 pandemic and governments’ response lead to dramatical change in quality of life worldwide. However, the extent of this change in Vietnamese medical students has not been documented.

## Objectives

The study aims to describe the quality of life and changes in quality of life of medical and nursing students during the COVID-19 pandemic and examine the association of quality of life and changes in quality of life with fear of COVID-19 and other socio-economic and demographic factors.

## Methods

The study was a cross-sectional study on all students of Hanoi Medical University from 3 majors: General Medicine, Preventive Medcine, Nursing; from 7th to 29th of April 2020; using an online questionnaire that included demographic and academic information, the

Vietnamese version of the SF-36 Quality of Life questionnaire and the Fear of COVID-19 Scale (FCV-19S). Linear and modified Poisson regression was used to examine the association between quality of life, changes in quality of life and other factors.

## Results

The number of participants was 1583 (response rate 43%). Among 8 dimensions of the SF36 (ranged 0-100), Vitality had the lowest score with a median score of 46. The median physical composite score (PCS) of the sample was 40.6 (IQR:20.8–53.2), 33.5% of the sample had an above-population average PCS score. The median mental composite score (MCS) of the sample was 20.3 (IQR:3.8–31.7), and 98.2% had an MCS score below average. 9.9% (95%CI:8.5%–11.4%) of the population reported a significant negative change in the quality of life. Fear of COVID-19 was not associated with significant changes in quality of life, nor MCS while having some association with PCS (Coef:-5.39;95%CI:-3to-7.8).

Perceived reduction in quality of life was also associated with: being on clinical rotation

COVID-19 (PR:1.5;95%CI:1.05–2.2), difficulties affording health services

(PR:1.4;95%CI:1.02–1.95), obesity (PR:2.38;95%CI:1.08–5.25) and chronic disease (PR:1.92;95%CI:1.23–3), typical symptoms (PR:1.85; 95%CI:1.23–2.78) and atypical symptoms of COVID-19 (PR:2.32;95%CI:1.41–3.81).

## Conclusion

The majority of medical and nursing students had below average quality of life, with lower vitality and mental composite health score in the settings of COVID-19. Perceived decrease in quality of life was associated with clinical rotation, difficulties affording healthcare services and was not associated with Fear of COVID-19.

Key word: quality of life, SF36, medical and nursing student, Covid-19

# Introduction

The COVID-19 pandemic caused by the novel coronavirus SARS-CoV-2, emerged in Wuhan, China in December 2019 [1]. Since then, the outbreak had progressed rapidly across the globe with a high death count and devastating impact on multiple aspects of life in many countries, including physical and mental health, politics, economy, and many other social issues[2–8]. Healthcare workers were shown to have a significantly higher risk of COVID19 infection, compared to the general public, and faced with extraordinary amounts of pressures leading to physical and mental exhaustion [9–12]. Medical and nursing students, especially those that are on clinical rotation, are being exposed to a similar environment and risk for COVID-19 infection.

Medical and nursing students, before the pandemic, were already known to face multiple physical and mental problems including burnout, anxiety, depression, and other mental health issues [13–15]. During the COVID-19 pandemic, students at higher education institutions, including medical students, greatly suffer from increased depressive symptoms, anxiety and sleep disturbance [16,17]. Medical education also changed rapidly in response to the situation, with the replacement of in-person classes with online equivalents and disruption of clinical rotations [18–20]. In Vietnam and many other countries, medical and nursing students also serve in the front line, supporting attempts to control the disease including contact tracing, providing counselling via telephone hotline, data management, cleaning/disinfecting, and taking test samples from suspected cases [21]. Together with other socio-economic aspects related to the pandemic like lockdown, social distancing, limitation in physical activities, the physical and mental quality of life of medical students are at high risk and can potentially lead to poorer health outcomes.

Understanding changes in medical students' quality of life is necessary to implement coping strategies and policies to reduce the burden that medical students are facing in the context of COVID-19. Therefore, we conducted this study to (1) describe the quality of life of medical and nursing students during the social distancing period and (2) examine the association between quality of life; fear of covid and other socioeconomics and demographics characteristics.

# Methods

## Study design and setting

A cross-sectional study was conducted on 1583 students major in medicine, preventive medicine, and nursing, at Hanoi Medical University, during the first 6 months of the covid19 pandemic in Vietnam, from 7th to 29th of April 2020.

Hanoi Medical University is one of the largest and most prestigious public medical schools in Vietnam. Every year, the enrolments for each major are about 400-500 for the general medicine program, 80 for the preventive medicine program and 140 for the nursing program. Students studying medicine and preventive medicine have a 6-year curriculum and are trained in general medicine (for general medicine), in preventive medicine (for preventive medicine) after they graduated, while nursing programs have a 4-year curriculum. Students studying general medicine and preventive medicine start their clinical rotation in the 2^nd^ semester of their third year, whereas nursing student start their rotation in the second semester of their second year. The detailed information on Vietnamese medical and nursing education can be found in other articles [22,23].

During first 6 months of the covid-19 pandemic in 2020, Vietnam underwent a temporary shutdown and implemented a social distancing policy [24–28]. The disease was being relatively controlled as the number of cases and mortality rate were rather small [24–27,29]. However, due to the policies, most conventional medical education activities, including clinical rotation were stopped and replaced with online education.

## Survey instruments

The questionnaire included questions regarding *demographic information*, *academic information* and *health information*, *the Fear of Covid 19 Scale (FCV-19S)*, and the *Quality of life SF-36 version 2.0 (SF-36)*, and *a question asking about the change in the general quality of life before and during the pandemic*.

The demographic information included: age, gender (Male/Female), marital status

(Married/Single), perceived affordability of healthcare services (No difficulties/Difficulties).

The academic information included: major (Doctor of Medicine/Doctor of Preventive Medicine/Nursing), academic year (First year/Second year/Third year/Fourth year/Fifth year/Sixth year), currently being on clinical rotation (Yes/No). Finally, the health information included: BMI (kg/m^2^), history of chronic disease (Yes/No) and current symptoms of COVID-19 (no symptoms, typical symptoms, atypical symptoms).

In our study, *the symptoms of COVID-19* were divided into 3 categories: no symptoms, typical symptoms, atypical symptoms; with typical symptoms being: fever, cough and dyspnea and atypical symptoms being fatigue, muscle aches, sputum, anxiety, headache, sore throat, congestion, chest pain, hemorrhage, nausea and vomiting. At the time of data collection, this categorization was used in Vietnam by the Ministry of Health to screen for COVID-19 [30]. BMI was classified into 4 categories according to WHO Asian – Pacific cutoff point: underweight (<18.5 kg/m2), normal weight (18.5–22.9 kg/m2), overweight (23–

24.9 kg/m2), and obese (≥25 kg/m2) [31].

*Fear of Covid-19* was assessed using the FCV-19S questionnaire. The FCV-19S questionnaire was translated into Vietnamese in a previous study and showed good itemscale convergent validity (mean of Rho = 0.77), discriminant validity and construct validity and high internal consistency (Cronbach’s alpha = 0.90) with the Vietnamese translation [32,33]. It consisted of 7 items and utilized a 5-point Likert scale with 1 = “strongly disagree”, 2 = “disagree”, 3 = “neither disagree or agree”, 4 = “agree”, 5 = “strongly agree”. The total score ranged from 7 to 35, with a higher score indicating greater fear of COVID19. We used a cut-off point of 21 with scores ranging from 7-20 categorized “Low FCV-19S score”, implying lower fear of COVID-19 and scores ranging from 21-35 categorized “High FCV-19S score”, implying higher fear of COVID-19 as we assumed that those who answered neutrally at every question would be having a score of 21.

The *quality of life of students* during the period was determined using the Vietnamese version of the Quality-of-life SF-36 version 2.0. The SF-36 determine the quality of life using 8 dimensions: Physical functioning (PF), Role limitations due to physical health (RP),

Role limitations due to emotional problems (RE), Vitality (VT), Emotional well-being (MH), Social functioning (SF), Pain (BP), General health (GH). The score for the 8 dimensions of the SF-36 ranged from 0-100 and positively correlate with the state of quality of life. A physical composite score (PCS) and mental composite score (MCS) were calculated from the

8 dimensions using a population-based scoring method [34,35]. The two summary scores, PCS and MCS, use the sum of the eight dimension z-scores derived from a reference population of Vietnamese people in the study by Watkins in 2000 [36], weighted by factor score coefficients derived from US 1990 general population estimates [34]. A PCS/MCS score of 50 represents the reference population average, any score below 50 would be considered “Below the population average”. The PCS and MCS scores with z-score derived from USA population averages is also included in the supplemental documents **(S8 Table)**. The Vietnamese version of the SF-36 was validated and was shown to provide a valid assessment of self-reported health status among the Vietnamese population, with all but two of the 36 items displayed good discriminant validity and all eight scale had good discriminant validity displayed the internal consistency of the 8 scales: PF (0.82), RP (0.86), BP (0.58), GH (0.66), VT(0.56), SF (0.67), RE (0.70), MH (0.55) [36].

*Perceived reduction in quality of life* was evaluated with a question asking the students

“How did your quality of life change compared to before the pandemic?” with the answer either “Worse” “Better” or “No difference”. Students that answered “Worse” were categorized as having their quality of life worsen while other students were categorized as not having no change or improvement in their quality of life.

## Sample size and data collection

The required sample size was estimated by using the formula for estimating sample mean [37], as follows:

213 With, n being the required sample size, Z being the standard error associated with the chosen
214 level of confidence 5%, σ being the standard deviation of the general quality of life score
215 taken from the population used to validate the Vietnamese version of the SF-36 [36].

From April 7th to 29th, 2020, all student (3672 students) from the Doctor of General

Medicine, Doctor of Preventive Medicine, and Bachelor of Nursing at Hanoi Medical

University were invited to participate in this survey. 1032 students from the Doctor of

General Medicine program (2921 invited, response rate 35.3%), 308 from the Doctor of Preventive Medicine program (466 invited, response rate 66.1%), and 243 from the Bachelor of Nursing program (285 invited, response rate 85.3%). agreed to participate in the study and completed the research questionnaire (1583/3672, resulting in a response rate of 43.1%.)

We collected data using an self-reported, online Google Form questionnaire, that included the study instruments. The research staff asked the Office for Student Services to send an official university notice with the questionnaire link to representatives of all invited classes.

Each representative then delivered the documents to their class’s social media groups. Thorough these channels, all students in invited classes could read the information regarding the study and decide whether to participate and complete the survey. The research staff also checked the responses and sent a reminder to classes with low response rate every week. At the end of April 2020, we closed the questionnaire link and extracted the data to an Excel file, which was then converted into a Stata data file.

## Data analysis

Stata 15.1 was used to analyze data. The Chi-squared, Fisher exact, t-test and Kruskal-Wallis test were used to compare the difference between groups of students regarding demographic characteristics, quality of life and changes in quality of life of participants. We consider ap value < 0.05 as statistically significant for all statistical tests.

Linear regression was used to determine the association between PCS and MCS score with demographic characteristics and fear of COVID-19 in students. Residual plots were used to check for linear model assumptions. The prevalence of people with changes in quality of life was high, thus, using logistic regression analysis to determine the association between the decrease in quality of life and independent variables may result in an overestimation of the relationship [38,39]. Therefore, we used a modified Poisson regression model with a robust error variance with binary data to directly calculate the Prevalence ratio (PR) as an appropriate alternative [38,39].

## Ethical issue

Our survey was approved by the Institutional Review Board of Hanoi Universities of Public Health and Hanoi Medical University (IRB No. 133/2020/YTCC-HD3). The online questionnaire provided participants with comprehensive information about the study. All participants gave informed consent by clicking “I agree to participate” box on the informed consent page. This consent procedure was approved by the IRB. Moreover, the participants could stop participating and close the Google Form at any time during the survey.

# Results

## Sample characteristics

The response rate for the study was 43% (1583/3672). The cohort characteristics are presented in **Table 1.** Of the 1583 participants, 65% were studying General Medicine, 19.5% were studying Preventive Medicine and 15.4% majored in Nursing. The proportion of participants currently on clinical rotation was 65.4%. There are several differences among the three cohorts regarding gender, age, affordability of healthcare services, BMI, as well as the prevalence of symptoms of COVID-19 and FCV-19S scores. The proportion of females was greater among Nursing (96.7%) and Preventive Medicine (75%) students than General Medicine (50.9%) students (p<0.001). Among nursing students, typical symptoms of COVID-19 were more prevalent at around 17.7% (p<0.001), compared to 10.7% in general medicine and 15.9% in preventive medicine. Additionally, nursing major students also had higher scores in the FCV-19S (Median: 19.00; IQR: 15.00 - 21.00; p<0.01), and had more students scored “High” on FCV-19S categories (44.4%, p<0.01), compared to students from other majors.

### Table 1. Sample characteristics

| Columns by: Academic majors | **General Medicine** | **Preventive Medicine** | **Nursing** | **Total** | **P-value** |
| --- | --- | --- | --- | --- | --- |
| n (%) | 1032 (65.2) | 308 (19.5) | 243 (15.4) | 1583 (100.0) |  |
| **Age, mean (sd)** | 21.74 (1.95) | 22.53 (1.83) | 20.10 (1.47) | 21.64 (2.00) | **<0.01** |
| **Gender, n (%)** |  |  |  |  |  |
| Female, n (%) | 525 (50.9) | 231 (75.0) | 235 (96.7) | 991 (62.6) |  |
| Male, n (%) | 507 (49.1) | 77 (25.0) | 8 (3.3) | 592 (37.4) | **<0.01** |
| **Academic years, n (%)** |  |  |  |  |  |
| First year, n (%) | 147 (14.2) | 38 (12.3) | 97 (39.9) | 282 (17.8) |  |
| Second year, n (%) | 247 (23.9) | 19 (6.2) | 79 (32.5) | 345 (21.8) |  |
| Third year, n (%) | 153 (14.8) | 36 (11.7) | 37 (15.2) | 226 (14.3) |  |
| Fourth year, n (%) | 103 (10.0) | 54 (17.5) | 30 (12.3) | 187 (11.8) |  |
| Fifth year, n (%) | 122 (11.8) | 74 (24.0) | 0 (0.0) | 196 (12.4) |  |
| Sixth year, n (%) | 260 (25.2) | 87 (28.2) | 0 (0.0) | 347 (21.9) | **<0.01** |
| **Currently on clinical rotation, n (%)** |  |  |  |  |  |
| No, n (%) | 394 (38.2) | 57 (18.5) | 97 (39.9) | 548 (34.6) |  |
| Yes, n (%) | 638 (61.8) | 251 (81.5) | 146 (60.1) | 1035 (65.4) | **<0.01** |
| **Marital status, n (%)** |  |  |  |  |  |
| Single, n (%) | 1026 (99.4) | 304 (98.7) | 242 (99.6) | 1572 (99.3) |  |
| Married, n (%) | 6 (0.6) | 4 (1.3) | 1 (0.4) | 11 (0.7) | 0.35 |
| **Affordability of healthcare services, n (%)** |  |  |  |  |  |
| No Difficulties, n (%) | 543 (52.6) | 179 (58.1) | 101 (41.6) | 823 (52.0) |  |
| Difficulties, n (%) | 489 (47.4) | 129 (41.9) | 142 (58.4) | 760 (48.0) | **<0.01** |
| **BMI categories, n (%)** |  |  |  |  |  |
| Underweight, n (%) | 168 (16.3) | 75 (24.4) | 79 (32.5) | 322 (20.3) |  |
| Normal, n (%) | 682 (66.1) | 191 (62.0) | 154 (63.4) | 1027 (64.9) |  |
| Overweight, n (%) | 163 (15.8) | 38 (12.3) | 8 (3.3) | 209 (13.2) |  |
| Obese, n (%) | 19 (1.8) | 4 (1.3) | 2 (0.8) | 25 (1.6) | **<0.01** |
| **Having chronic disease, n (%)** |  |  |  |  |  |
| No, n (%) | 961 (93.1) | 281 (91.2) | 226 (93.0) | 1468 (92.7) |  |
| Yes, n (%) | 71 (6.9) | 27 (8.8) | 17 (7.0) | 115 (7.3) | 0.53 |
| **Symptoms of Covid-19, n (%)** |  |  |  |  |  |
| No symptoms, n (%) | 866 (83.9) | 244 (79.2) | 172 (70.8) | 1282 (81.0) |  |
| Typical Symptoms, n (%) | 110 (10.7) | 49 (15.9) | 43 (17.7) | 202 (12.8) |  |
| Atypical Symptoms, n (%) | 56 (5.4) | 15 (4.9) | 28 (11.5) | 99 (6.3) | **<0.01** |
| **FCV-19S score categories, n (%)** |  |  |  |  |  |

| Low, n (%) | 756 (73.3) | 221 (71.8) | 135 (55.6) | 1112 (70.2) |  |
| --- | --- | --- | --- | --- | --- |
| High, n (%) | 276 (26.7) | 87 (28.2) | 108 (44.4) | 471 (29.8) | **<0.01** |
| **FCV-19S score, median (iqi)** | 16.00 (13.00; 21.00) | 16.00 (12.25; 21.00) | 19.00 (15.00; 21.00) | 16.00 (13.00; 21.00) | **<0.01** |
|  |  |  |  |  |  |
| Statistical comparison Statistical comparison using Chi-square test for categorical variable - display as n(%);T test for continuous-normally distributed variable - display as mean(sd); Wilcoxon rank-sum test for continuous-skewed variable - display as median(iqi); The bold p-value indicated statistical significance (p<0.05). | | | | | |

268

## 269 Description of the SF-36 scores

270 **Table 2** describe the median (IQR) the scoring on PCS, MCS and 8 dimensions of the SF-36
271 in different groups

### 272 Table 2. PCS, MCS, and scoring of the 8 dimensions of the SF-36

|  | **Physical Composite Score (PCS)** | **Mental**  **Composite Score (MCS)** | **Physical functioning (PF)** | **Role limitations due to physical health (RP)** | **Role limitations due to emotional problems (RE)** | **Vitality (VT)** | **Emotional well-being**  **(MH)** | **Social functioning (SF)** | **Pain (BP)** | **General health (GH)** |
| --- | --- | --- | --- | --- | --- | --- | --- | --- | --- | --- |
| Total | 40.6 (20.8 - 53.2) | 20.3 (3.8 - 31.7) | 90 (70 - 100) | 100 (50 - 100) | 100 (33.3 - 100) | 46 (36 - 56) | 64 (52 - 76) | 75 (62.5 - 87.5) | 100 (80 - 100) | 71.3 (58.8 - 77.5) |
| **Academic majors** | |  |  |  |  |  |  |  |  |  |
| General Medicine | 43.2 (22.7-53.8) | 20.1 (3.3-31.3) | 90 (70-100) | 100 (50-100) | 100 (33.3-100) | 46 (36-56) | 64 (52-76) | 75 (62.5-87.5) | 100 (80-100) | 71.3 (58.8-77.5) |
| Preventive Medicine | 39.9 (21.3-52.5) | 22.5 (8-34.4) | 90 (70-100) | 100 (50-100) | 100 (33.3-100) | 46 (36.3-56.3) | 64 (52-76) | 75 (62.5-100) | 100 (80-100) | 71.3 (58.8-81.3) |
| Nursing | 29.3 (12.5-48.6) | 17.3 (2.8-29.9) | 85 (60-95) | 75 (25-100) | 100 (33.3-100) | 46 (36-56) | 60 (52-72) | 62.5 (50-87.5) | 100 (80-100) | 71.3 (58.8-77.5) |
| P value | **<0.001** | **0.009** | **0.001** | **<0.001** | **0.026** | 0.474 | **0.004** | **<0.001** | **0.007** | 0.417 |
|  |  |  |  |  |  |  |  |  |  |  |
| **Gender** | |  |  |  |  |  |  |  |  |  |
| Female | 38.8 (20.8-51.9) | 19.8 (3.7-31.4) | 90 (70-100) | 100 (50-100) | 100 (33.3-100) | 46 (36-56) | 64 (52-72) | 75 (62.5-87.5) | 100 (80-100) | 71.3 (58.8-77.5) |
| Male | 45.1 (21.2-54.2) | 21.1 (4.3-32.2) | 95 (65-100) | 100 (50-100) | 100 (66.7-100) | 46 (38.5-56.3) | 64 (52-76) | 75 (62.5-87.5) | 100 (80-100) | 71.3 (58.8-81.3) |
| P value | **0.005** | 0.288 | **0.004** | **0.031** | 0.077 | 0.105 | 0.250 | 0.375 | **0.004** | 0.417 |
|  |  |  |  |  |  |  |  |  |  |  |
| **Academic years*** | |  |  |  |  |  |  |  |  |  |
| First year | 27.8 (11.1-44.1) | 16.6 (0.7-32.5) | 80 (55-95) | 75 (25-100) | 66.7 (33.3-100) | 46.3 (36-56) | 64 (52-72) | 62.5 (50-87.5) | 100 (80-100) | 71.3 (58.8-77.5) |
| Second year | 39.3 (18.1-51.5) | 20.3 (5.5-31.8) | 90 (65-100) | 100 (50-100) | 100 (66.7-100) | 46 (36.3-51.5) | 60 (52-72) | 75 (62.5-87.5) | 100 (80-100) | 71.3 (58.8-77.5) |
| Third year | 39.5 (20.7-53.2) | 19.9 (3.2-29) | 90 (70-100) | 100 (50-100) | 100 (66.7-100) | 45.8 (36-51) | 60 (52-68) | 75 (62.5-87.5) | 100 (80-100) | 65 (52.5-77.5) |
| Fourth year | 47.6 (30.2-54.3) | 21.5 (6.8-32.4) | 95 (85-100) | 100 (75-100) | 100 (66.7-100) | 46 (36-56.3) | 60 (52-76) | 75 (62.5-87.5) | 100 (90-100) | 65 (58.8-77.5) |
| Fifth year | 46.7 (31.3-55.7) | 23 (8.7-32.3) | 95 (80-100) | 100 (100-100) | 100 (66.7-100) | 46 (40.8-56.3) | 68 (52-76) | 75 (62.5-100) | 100 (80-100) | 71.3 (58.8-83.8) |
| Sixth year | 44.8 (25-54.2) | 20.6 (0.3-33.2) | 95 (75-100) | 100 (50-100) | 100 (33.3-100) | 50.3 (36-56.3) | 68 (56-80) | 75 (62.5-87.5) | 100 (90-100) | 71.3 (58.8-83.8) |
| P value | **<0.001** | 0.247 | **<0.001** | **<0.001** | **<0.001** | **<0.001** | **<0.001** | **<0.001** | **<0.001** | **<0.001** |
|  |  |  |  |  |  |  |  |  |  |  |
| **Currently on clinical rotation** | |  |  |  |  |  |  |  |  |  |
| No | 32.4 (13.6-49.4) | 18.4 (2.8-31.8) | 85 (55-95) | 87.5 (25-100) | 100 (33.3-100) | 46 (36-56) | 60 (52-72) | 75 (62.5-87.5) | 100 (80-100) | 71.3 (58.8-77.5) |
| Yes | 44.5 (24.1-54.1) | 21 (5.2-31.6) | 95 (75-100) | 100 (50-100) | 100 (33.3-100) | 46 (36-56) | 64 (52-76) | 75 (62.5-87.5) | 100 (80-100) | 71.3 (58.8-81.3) |
| P value | **<0.001** | 0.316 | **<0.001** | **<0.001** | **<0.001** | 0.295 | **0.029** | **<0.001** | **0.001** | 0.692 |
|  |  |  |  |  |  |  |  |  |  |  |
| **Marital status** | |  |  |  |  |  |  |  |  |  |
| Single | 40.6 (20.8-53.2) | 20.3 (4-31.7) | 90 (70-100) | 100 (50-100) | 100 (33.3-100) | 46 (36-56) | 64 (52-76) | 75 (62.5-87.5) | 100 (80-100) | 71.3 (58.8-77.5) |
| Married | 49.9 (23.1-52.7) | 13.8 (-1.9-37.7) | 95 (75-100) | 100 (50-100) | 100 (33.3-100) | 51 (35.8-56.3) | 72 (60-84) | 75 (62.5-75) | 100 (100-100) | 77.5 (58.8-77.5) |
| P value | 0.514 | 0.735 | 0.343 | 0.978 | 0.841 | 0.867 | 0.207 | 0.837 | 0.227 | 0.632 |
|  |  |  |  |  |  |  |  |  |  |  |
| **Affordability of healthcare services** | | | | | |  |  |  |  |  |
| No Difficulties | 44.1 (25.7-53.9) | 23.5 (8.3-33.7) | 90 (75-100) | 100 (75-100) | 100 (66.7-100) | 50.3 (40.8-56.3) | 68 (56-76) | 75 (62.5-87.5) | 100 (80-100) | 71.3 (58.8-83.8) |
| Difficulties | 36.4 (15.3-52.1) | 17.1 (-0.7-29.5) | 85 (65-100) | 100 (25-100) | 100 (33.3-100) | 45.5 (36-51.3) | 60 (52-72) | 75 (50-87.5) | 100 (80-100) | 65 (52.5-77.5) |
| P value | **<0.001** | **<0.001** | **<0.001** | **<0.001** | **<0.001** | **<0.001** | **<0.001** | **<0.001** | **<0.001** | **<0.001** |
|  |  |  |  |  |  |  |  |  |  |  |
| **BMI categories** | | | | | |  |  |  |  |  |
| Underweight | 36.4 (16.5-50.7) | 18.7 (2.3-29.9) | 85 (65-100) | 100 (50-100) | 100 (33.3-100) | 45.5 (36-51.3) | 60 (52-72) | 75 (62.5-87.5) | 100 (80-100) | 65 (52.5-77.5) |
| Normal | 41.6 (21.6-53.8) | 20.7 (4.9-32) | 90 (70-100) | 100 (50-100) | 100 (33.3-100) | 46 (36.3-56) | 64 (52-76) | 75 (62.5-87.5) | 100 (80-100) | 71.3 (58.8-83.8) |
| Overweight | 44 (25.4-53.5) | 21.3 (3.2-32.9) | 90 (70-100) | 100 (75-100) | 100 (66.7-100) | 46.3 (40.5-56.3) | 64 (56-76) | 75 (62.5-87.5) | 100 (90-100) | 71.3 (58.8-81.3) |
| Obese | 39.3 (21.9-51.3) | 18.2 (1.6-36.6) | 90 (80-95) | 100 (25-100) | 100 (33.3-100) | 46 (36-56.3) | 72 (56-84) | 62.5 (50-87.5) | 100 (80-100) | 71.3 (58.8-77.5) |
| P value | **0.007** | 0.471 | 0.053 | 0.117 | 0.270 | **0.005** | **0.001** | 0.719 | 0.054 | **<0.001** |
|  |  |  |  |  |  |  |  |  |  |  |
| **Symptoms of Covid-19** | | | | | |  |  |  |  |  |
| No symptoms | 43.2 (22.6-53.9) | 21 (5.8-32.2) | 90 (70-100) | 100 (50-100) | 100 (33.3-100) | 46 (36.3-56.3) | 64 (52-76) | 75 (62.5-87.5) | 100 (80-100) | 71.3 (58.8-81.3) |
| Atypical symptoms | 23.1 (6.1-42.1) | 10.9 (-7-27) | 85 (60-95) | 75 (25-100) | 66.7 (0-100) | 40.8 (36-46.3) | 56 (48-68) | 62.5 (50-75) | 80 (60-100) | 58.8 (46.3-71.3) |
| Typical symptoms | 35.3 (17.2-49) | 17.6 (-1-30.9) | 90 (70-95) | 75 (25-100) | 100 (33.3-100) | 45.8 (36-55.5) | 64 (52-76) | 75 (62.5-87.5) | 100 (80-100) | 68.1 (58.8-77.5) |
| P value | **<0.001** | **<0.001** | **<0.001** | **<0.001** | **<0.001** | **<0.001** | **<0.001** | **<0.001** | **<0.001** | **<0.001** |
|  |  |  |  |  |  |  |  |  |  |  |
| **Having chronic disease** | | | | | |  |  |  |  |  |
| No | 40.7 (20.9-53.2) | 20.7 (5.1-31.9) | 90 (70-100) | 100 (50-100) | 100 (33.3-100) | 46 (36-56) | 64 (52-76) | 75 (62.5-87.5) | 100 (80-100) | 71.3 (58.8-81.3) |
| Yes | 40.3 (19.6-51) | 14.5 (-4.5-27.8) | 90 (70-100) | 100 (50-100) | 66.7 (0-100) | 46 (36-51.5) | 64 (52-72) | 75 (62.5-87.5) | 100 (70-100) | 65 (52.5-77.5) |
| P value | 0.457 | **0.007** | 0.621 | 0.257 | **0.001** | 0.111 | 0.312 | 0.484 | **0.021** | **0.011** |
|  |  |  |  |  |  |  |  |  |  |  |
| **FCV-19S score categories** | | | | | |  |  |  |  |  |
| Low | 43.6 (23.5-53.9) | 21.2 (3.9-32.9) | 90 (75-100) | 100 (50-100) | 100 (33.3-100) | 46.3 (40.8-56.3) | 68 (56-76) | 75 (62.5-87.5) | 100 (80-100) | 71.3 (58.8-83.8) |
| High | 31.7 (11.2-49.8) | 18.6 (3.8-29) | 85 (55-100) | 100 (25-100) | 100 (33.3-100) | 41 (36-51.3) | 56 (52-68) | 75 (62.5-87.5) | 100 (80-100) | 65 (52.5-77.5) |
| P value | **<0.001** | **<0.001** | **<0.001** | **0.023** | 0.663 | **<0.001** | **<0.001** | **0.001** | **<0.001** | **<0.001** |
|  |  |  |  |  |  |  |  |  |  |  |
| **How have your quality of life changed compare to before the pandemic** | | | | | |  |  |  |  |  |
| Not Worsen | 41.9 (22.5-53.3) | 21.5 (7.4-32.5) | 90 (70-100) | 100 (50-100) | 100 (66.7-100) | 46 (36.3-56.3) | 64 (52-76) | 75 (62.5-87.5) | 100 (80-100) | 71.3 (58.8-81.3) |
| Worsen | 23.9 (3.5-45.4) | -3.5 (-14.3-17.7) | 85 (55-95) | 37.5 (0-100) | 33.3 (0-66.7) | 36.3 (31.3-46) | 58 (48-70) | 62.5 (50-75) | 90 (70-100) | 58.8 (52.5-71.3) |
| P value | **<0.001** | **<0.001** | **<0.001** | **<0.001** | **<0.001** | **<0.001** | **<0.001** | **<0.001** | **<0.001** | **<0.001** |
| All data was presented as median (IQI). Statistical comparison using:  Wilcoxon rank-sum test for continuous-skewed variable - display as median(iqr) for comparison between 2 groups; Kruskal-Wallis test for continous-skewed variables - display as median (iqr) for comparison between 3 or more groups; Bold p-value indicated statistical significance (p<0.05).  *Fifth and sixth year students are only from General Medicine and Preventive Medicine major | | | | | |  |  |  |  |  |

273

### Eight dimensions of the SF-36

The median score for the dimension of the SF-36 were PF = 90, RP = 100, RE = 100, VT = 46, MH = 64, SF = 75, BP = 100, and GH = 71.3 with **Table 2** described the 8 dimensions of

SF-36 score by major. There was no difference in VT, GH between majors and gender.

However, nursing students have lower PF, RP, RE, MH, SF, BP scores compared to

Preventive Medicine and General Medicine students (p<0.001). Females have lower physical functioning (PF), more role limitation due to physical functioning (RP) and bodily pain (BP) compared to males.

### Physical Composite Score

The median PCS score of the sample was 40.6 (IQR: 20.8 – 53.2). There was a significant difference in the PCS score between the 3 majors (p<0.001)**.** The PCS score of general medicine, preventive medicine and nursing students were 43.2 (IQR: 22.7-53.8), 39.9 (IQR: 21.3-52.5) and 29.3 (IQR: 12.5-48.6) respectively. PCS score was also higher in males than females (p=0.005), in students in later years of the program (from 4^th^ year) than those in earlier years (p<0.001), in students on clinical rotation than those not on rotation (p<0.001), and in students who had no difficulties affording healthcare services than those with difficulties (p<0.001). Compared to normal students, the PCS score was significantly lower in those who have symptoms of COVID-19 (p <0.001) and those who have significant Fear of COVID-19 (p <0.001). There was no difference in PCS scores regarding chronic diseases status. Overall, 66.5% (95% CI: 64.1% – 68.8%) of the sample had PCS score below the reference population average. Nursing major has the highest number of students who had a below average score (77.4%; p <0.01) (**S1 and S2 Tables)**

### Mental Composite Score

The median MCS score of the sample was 20.3 (IQR: 3.8 - 31.7). Similar to the PCS score, there was a difference between 3 majors (p<0.001) with the MCS score of 20.1 (IQR: 3.3 - 31.3) for general medicine, 22.5 (IQR: 8 - 34.4) for preventive medicine and 17.3 (IQR: 2.8 - 29.9) for nursing. The MCS score was also higher in students who had no difficulties affording healthcare services than those with such difficulties (p<0.001), in those who did not have symptoms of COVID-19 than those with symptoms (p<0.001), and in those who had insignificant fear of COVID-19 than those with significant fear (p<0.001). Students having chronic disease also had a lower MCS score, compared to those who did not (p=0.007). On the other hand, there was no difference in the MCS score regarding gender, academic year, clinical rotation. Compared to the reference population average, 98.2% (97.5

– 98.8) of the sample have a lower MCS score (**S1 and S2 Tables)**

## Regression Analysis of PCS and MCS scores

We noticed no specific pattern in the residual analysis of the two linear regression models of PCS and MCS score **(S3-S4 Figs).**

### Table 3. Linear regression of PCS score

|  | **Physical Composite Score** | | | |  |
| --- | --- | --- | --- | --- | --- |
|  | **Coef.** | **p-value** | | **95%** | **CI** |
| **Academic Major** |  |  | |  |  |
| General Medicine | ref. |  | |  |  |
| Preventive Medicine | -3.31 | **0.024** | | -6.2 | -0.43 |
| Nursing | -5.67 | **0.001** | | -9 | -2.4 |
| **Gender** |  |  | |  |  |
| Female | ref. |  | |  |  |
| Male | -0.62 | 0.629 | | -3.1 | 1.9 |
| **Currently on Clinical Rotation** | |  | |  |  |
| No | ref. |  | |  |  |
| Yes | 8.89 | **<0.001** | | 6.6 | 11 |
| **Marital Status** |  |  | |  |  |
| Single | ref. |  |  |  |  |
| Married | 3.35 |  | 0.612 | -9.6 | 16 |
| **Affordabilities of Healthcare Services** | | |  |  |  |
| No Difficulties | ref. |  |  |  |  |
| Difficulties | -6.32 |  | **<0.001** | -8.5 | -4.1 |
| **BMI Category** |  |  |  |  |  |
| Underweight | ref. |  |  |  |  |
| Normal | 2.28 |  | 0.108 | -0.5 | 5.1 |
| Overweight | 3.44 |  | 0.095 | -0.59 | 7.5 |
| Obese | -1 |  | 0.828 | -10 | 8 |
| **Having Chronic Disease** |  |  |  |  |  |
| No | ref. |  |  |  |  |
| Yes | -0.35 |  | 0.868 | -4.5 | 3.8 |
| **Symptoms of Covid-19** |  |  |  |  |  |
| No symptoms | ref. |  |  |  |  |
| Typical Symptoms | -4.96 |  | **0.003** | -8.2 | -1.7 |
| Atypical Symptoms | -11.52 |  | **<0.001** | -16 | -7 |
| **Fear of Covid Scale score categories** | | |  |  |  |
| Low | ref. |  |  |  |  |
| High | -5.39 |  | **<0.001** | -7.8 | -3 |

There was no difference in PCS scores between gender after accounting for other sociodemographic and academic characteristics, the FCV-19S scale, and symptoms of COVID-19. Chronic disease was also not associated with PCS score. Lower PCS score was associated with: majoring in Preventive Medicine (Coef: -3.3; 95% CI: -6.2 to -0.43) or Nursing (Coef: -5.67; 95% CI: -9 to -2.4), significant fear of COVID-19 (Coef: -5.39; 95% CI: -3 to -7.8) and typical (Coef: -4.96; 95% CI: -8.2 to -1.7) and atypical (Coef: -11.52; 95% CI: -16 to 7) symptoms of COVID-19, difficulties affording healthcare services were associated with PCS (Coef: -6.32; 95% C: -8.5 to -4.1). Higher PCS score was associated with being on clinical rotation (Coef: 8.89; 95% CI: 6.6 to 11). The factors with the highest influence on PCS score was being on clinical rotation (Coef: 8.89; 95% CI: 6.6 to 11) and having atypical symptoms (Coef: -11.52; 95% CI: -16 to -7). An identical pattern of association was also found in our quantile regression analysis with same dependent and independent variables **(S5 Table)**, with the only difference being that Preventive Medicine major was not statistical significant associated with lower PCS score (Coef: -3.00; 95% CI: -6.8 to 0.79). **Table 4. Linear regression of MCS score**

|  | **Mental Composite Score** | | |  |
| --- | --- | --- | --- | --- |
|  | **Coef.** | **p-value** | **95%** | **CI** |
| **Academic Major** |  |  |  |  |
| General Medicine | ref. |  |  |  |
| Preventive Medicine | 2.99 | **0.028** | 0.33 | 5.6 |
| Nursing | 0.34 | 0.827 | -2.7 | 3.4 |
| **Gender** |  |  |  |  |
| Female | ref. |  |  |  |
| Male | 0.53 | 0.651 | -1.8 | 2.9 |
| **Currently on Clinical Rotation** |  |  |  |  |
| No | ref. |  |  |  |
| Yes | 1.29 | 0.238 | -0.85 | 3.4 |
| **Marital Status** |  |  |  |  |
| Single | ref. |  |  |  |
| Married | -1.06 | 0.862 | -13 | 11 |
| **Affordabilities of Healthcare Services** |  |  |  |  |
| No Difficulties | ref. |  |  |  |
| Difficulties | -5.2 | **<0.001** | -7.2 | -3.2 |
| **BMI Category** |  |  |  |  |
| Underweight | ref. |  |  |  |
| Normal | 0.64 | 0.622 | -1.9 | 3.2 |
| Overweight | 1.1 | 0.563 | -2.6 | 4.8 |
| Obese | 1.71 | 0.687 | -6.6 | 10 |
| **Having Chronic Disease** |  |  |  |  |
| No | ref. |  |  |  |
| Yes | -5.6 | **0.004** | -9.5 | -1.7 |
| **Symptoms of Covid-19** |  |  |  |  |
| No symptoms | ref. |  |  |  |
| Typical Symptoms | -2.38 | 0.125 | -5.4 | 0.66 |
| Atypical Symptoms | -7.59 | **<0.001** | -12 | -3.4 |
| **Fear of Covid Scale score categories** |  |  |  |  |
| Low | ref. |  |  |  |
| High | -0.95 | 0.4 | -3.2 | 1.3 |

There was also no difference in MCS scores between gender. Significant fear of COVID-19 and being on clinical rotation had no statistically significant effect on MCS score. Regarding academic characteristics, majoring in Preventive Medicine result in higher MCS score (Coef:

2.97; 95% CI: 0.33 to 5.6) compared to those who were majoring in general medicine and nursing. Lower MCS score was associated with: having chronic disease (Coef: -9.5; 95% CI: -1.7 to -5.6), having atypical symptoms of COVID-19 (Coef: -7.59; 95% CI: -12 to -3.4), difficulties affording healthcare services (Coef: -5.20; 95% CI: -7.2 to -3.2). The association of having atypical symptoms and affordability of healthcare services with MCS score was also consistent with our quantile regression analysis. However, in the quantile regression analysis with the same variables **(S6 Table)**, there were no association between the Preventive Medicine major (p=0.064), chronic disease and MCS (p=0.073), but being on clinical rotation was associated with a higher MCS score (Coef: 3.239; 95% CI: 0.71 to 5.8).

### Perceived negative changes in quality of life

Overall, 9.9% (95% CI: 8.5% – 11.4%) of the study population reported a significant negative change in quality of life. 20.2% of 6^th^-year medical students reported having a reduction in quality of life, a prevalence much higher than that of their junior (<10%) (p<0.01). Also, students that were on clinical rotation reported a negative changes in quality of life was higher (11.4%) compared to the who weren’t (6.9%) (p<0.01). A significant portion of those who were obese (28%) or having chronic disease (20.9%) perceived a decrease in the quality of life , while this decrease is much less prevalent in students with normal BMI (9.8%) or those without chronic disease (9.0%) (p<0.05). There were no significant differences among genders (p=0.25), marital status (p=0.35), academic majors, and FCV-19S score (p=0.08). **(S7 Table)**

**Table 5** shows the modified Poisson regression analysis of perceived negative changes in the quality of life of medical students. Results showed that being on clinical rotation was associated with more deterioration in quality of life during COVID-19 (PR: 1.5; 95% CI:

1.05 – 2.2). Additionally, students that had difficulties affording health services were more susceptible to these negative changes compared to those who don’t (PR: 1.4; 95% CI: 1.02 –

1. 95). Obesity (PR: 2.38; 95% CI: 1.08 – 5.25), having chronic disease (PR: 1.92; 95% CI:

1.23 – 3), and typical symptoms (PR: 1.85; 95% CI: 1.23 – 2.78), atypical symptoms of COVID-19 (PR: 2.32; 95% CI: 1.41 – 3.81) were also associated with the reduction in quality of life of the sample.

### Table 5. Poisson regression analysis of negative changes in quality of life

|  | **Changes in Quality of Life** | | | |
| --- | --- | --- | --- | --- |
|  | **PR** | **p-value** | **95%** | **CI** |
| **Academic Major** | |  |  |  |
| General Medicine | ref. |  |  |  |
| Preventive Medicine | 1.10 | 0.631 | 0.74 | 1.65 |
| Nursing | 0.92 | 0.742 | 0.55 | 1.53 |
| **Gender** |  |  |  |  |
| Female | ref. |  |  |  |
| Male | 1.22 | 0.282 | 0.85 | 1.76 |
| **Currently on Clinical Rotation** | | |  |  |
| No | ref. |  |  |  |
| Yes | 1.52 | **0.028** | 1.05 | 2.22 |
| **Marital Status** | |  |  |  |
| Single | ref. |  |  |  |
| Married | 1.57 | 0.529 | 0.39 | 6.42 |
| **Affordabilities of Healthcare Services** | | | |  |
| No Difficulties | ref. |  |  |  |
| Difficulties | 1.41 | **0.037** | 1.02 | 1.95 |
| **BMI Category** | |  |  |  |
| Underweight | 0.82 | 0.371 | 0.54 | 1.26 |
| Normal | ref. |  |  |  |
| Overweight | 0.85 | 0.522 | 0.52 | 1.40 |
| Obese | 2.38 | **0.032** | 1.08 | 5.25 |
| **Having Chronic Disease** | | |  |  |
| No | ref. |  |  |  |
| Yes | 1.92 | **0.004** | 1.23 | 3.00 |
| **Symptoms of Covid-19** | | |  |  |
| No symptoms | ref. |  |  |  |
| Typical Symptoms | 1.85 | **0.003** | 1.23 | 2.78 |
| Atypical Symptoms | 2.32 | **0.001** | 1.41 | 3.81 |
| **FCV-19S score categories** | | |  |  |
| Low | ref. |  |  |  |
| High | 0.71 | 0.078 | 0.49 | 1.04 |

### IV. Discussion

From our study, it can be seen that there is a similar pattern in quality of life between different groups of students: a lower score in vitality (VIT = 46), emotional functioning (MH = 64) and social functioning (SF = 75) with vitality being the lowest. This pattern is similar to a pattern from other studies on medical students using the SF-36 instruments, outside of the pandemic

[40,41]. A study on Italian medical students from 2005-2015 showed a similar pattern of lower VIT (Mean: 59.4; SD: 16.1), MH (Mean: 68.65; SD: 16.3) compared to other dimensions and reference Italian population [41]. Compared to the general population, medical students were already suffering from burnout, stress and multiple mental issues, as mentioned in previous studies in other countries [13–15,42]. Combined with sudden changes in the context of the pandemic, students are having symptoms of depression, anxiety, sleep disturbances [16,17,19,43]. Low vitality, fatigue, extensive feelings of sleepiness and increased daily nap time were recorded in multiple studies [43–45].

An extremely low level of mental quality of life was recorded as 98.2% of students have a lower MCS score (Median: 20.3 IQR: 3.8 - 31.7) than that of the reference population (MCS: 50) The extreme number could be partially explained due to the reference Vietnamese population being young with an average age of 27 and had a high quality of life, and the belowaverage medical students quality of life suggested in multiple studies outside of the pandemic [40,41]. However, the median MCS score was still exceptionally low and thus, the overall mental status of the students should be critically alarming. In our study, mental quality of life was found to be associated with academic major (Coef: 2.97; 95% CI: 0.33 to 5.6), affordabilities of healthcare services (Coef: -5.20; 95% CI: -7.2 to -3.2), chronic disease (Coef:

-9.5; 95% CI: -1.7 to -5.6), and atypical symptoms of COVID-19 (Coef: -7.59; 95% CI: -12 to -3.4), while not being associated with gender. The association of financial difficulties and quality of life is rather similar to the findings of a study on depression on 4^th^, 5^th^ and 6^th^-year students of Hanoi Medical University before the pandemic, in 2019 [42]. However, a study by Vo on medical students in southern Vietnam, in the same year 2019, shows that female has a lower mental and physical quality of life [46]. This variation in results could be explained by the difference in environmental exposure between the two cohorts as there are major differences in the curriculum and academic environments, as some southern medical schools used a organ-based and modular curriculum for general medicine program, while Hanoi Medical University still used a traditional discipline-based curriculum for for general medicine program at the time of this survey.

Regarding physical health, the PCS score of the sample has a median score of 40.6 with 66.5% below the population average. The low level of physical health is likely to be related to the low level of mental health, vitality and increased fatigue reduced physical activities during the lockdown [45,47–49]. There have been mixed findings regarding gender and physical-related quality of life around the world and Vietnam [46,50,51]. Similar to mental quality of life., physical quality of life was lower in females in a study on southern medical students of Vietnam [46]. However, this association was also not observed in our study. On the other hand, difficulties affording healthcare services (Coef: -6.32; 95% C: -8.5 to -4.1), fear of COVID19 (Coef: -5.39; 95% CI: -3 to -7.8), and symptoms of COVID-19: typical (Coef: -4.96; 95% CI: -8.2 to -1.7) and atypical (Coef: -11.52; 95% CI: -16 to -7) were found to be correlated with reduced physical health. In addition, academic major and clinical rotation also associated with physical health, as preventive medicine (Coef: -3.3; 95% CI: -6.2 to -0.43) and nursing students (Coef: -5.67; 95% CI: -9 to -2.4) have lower PCS scores and students being on clinical rotation have better physical health (Coef: 8.89; 95% CI: 6.6 to 11).

There was no relationship between genders and reduction in quality of life in our study, consistent with findings on PCS and MCS scores. Significant perceived negative changes in quality of life were associated with being on clinical rotation (PR: 1.5; 95% CI: 1.05 – 2.2), difficulties affording healthcare services (PR: 1.41; 95% CI: 1.02 – 1.95), obesity (PR: 2.38; 95% CI: 1.08 – 5.25), having chronic diseases (PR: 1.92; 95% CI: 1.23 – 3), and having symptoms of COVID-19: typical symptoms (PR: 1.85; 95% CI: 1.23 – 2.78), atypical symptoms of COVID-19 (PR: 2.32; 95% CI: 1.41 – 3.81). Despite having higher PCS and relatively the same MCS score, students on clinical rotation are more likely to perceive negative changes in their quality of life (PR: 1.5; 95% CI: 1.05 – 2.2), which is probably due to visible disruption in clinical rotation and medical rotation and concerns for their career development, which was explored in prior studies [17,19]. Students who have difficulties affording healthcare services are 1.41 times (PR: 1.41; 95% CI: 1.02 – 1.95) more likely to feel that their quality of life has worsened. Affordability of healthcare services consistently correlated with changes in quality of life and quality of life of students during the pandemic, as it could place an invisible pressure on the person should they get infected. According to our study, students who have obesity and/or chronic disease are more vulnerable to deterioration in their quality of life. Compared to a person with a normal BMI, an obese person is 2.38 times (PR: 2.38; 95% CI: 1.08 – 5.25) more likely to have worse quality of life in the pandemic, while having chronic diseases increases the prevalence 1.92 times (PR: 1.92; 95% CI: 1.23 – 3). An interesting finding of our study is that atypical symptoms had an impact on MCS scores

(Coef: -7.59; 95% CI: -12 to -3.4) while typical symptoms did not. The impact of atypical symptoms on mental quality of life could be due to the physical impairment it brings and the uncertainty in making the decision to get a COVID-19 test [52]. However, there was no association between Fear of COVID-19 and reduction in quality of life (PR: 0.71; 95% CI: 0.49 – 1.04). Therefore, we hypothesized that the symptoms of COVID-19 mostly affected medical students via physical impairment, rather than fear and anxiety because Fear of COVID-19 was not found to be associated with mental quality of life but had some relation to the physical quality of life. This result could be explained by the restrictions of physical activities during the lockdown, while the pandemic was relatively controlled. Therefore, Fear of COVID-19 could have a lesser impact than expected on medical students’ mental health and anxiety during the pandemic, as the change was primarily due to previously mentioned

factors.

### Strength and limitations

The strength of our study is that our represtative sample frame included all students from 3 majors: General Medicine, Preventive Medicine and Nursing. Moreover, the main instrument - the Vietnamese version of the SF-36 was validated and was shown to provide a valid assessment of self-reported health status among the Vietnamese population and allowed for both compound and specific evaluation of quality life. Finally, the survey was conducted after the first wave of COVID hit Vietnam - after Bach Mai hospital, a major teaching hospital of Hanoi Medical University, was locked down after a case series COVID-19 on March 28^th^, 2020. Therefore, we believe that the result captures the changes in quality of life of the students during the early stage of the pandemic. However, this study used an online, anonymous data collection scheme, which lead to a low response rate of only about 43%, and potential sampling errors, and selection bias. Moreover, there was no available data on medical students' quality of life using the same instruments prior the pandemic, so a direct comparison was not possible. The use of a cross-sectional study design also limited our interpretation of the results to association, rather than causation. This design was also unable to examine the changes in quality of life of the students in other waves of COVID-19 and their adaptation going further into the pandemic. Also, the translation into Vietnamse of the SF-36, FCV-19S might affect the validity of these questionnaire. Furthermore, the SF-36 tools scoring used populationbased scoring methods, however, there was no Vietnamese population-derived weighting coefficient for 8 dimensions of SF-36 when calculating PCS and MCS score, thus we had to use the coefficient from the original USA population, which can cause a slight alteration in the results, as the weight for each population is slightly different [34]. Additionally, since we only use a single direct question to examine the reduction in quality of life, it is difficult to accurately determine the specific changes in quality of life of healthcare students in the pandemic.

### V. Conclusion

The physical and mental quality of life of the sample was lower than the general population.

Vitality, mental health, and social functioning dimensions showed the lowest score among the 8 dimensions. Lower PCS score was associated with academic majors, chronic disease, not being on clinical rotation, difficulties affording healthcare services, and Fear of COVID-19. Lower MCS score was associated with academic major, difficulties affording healthcare services, and chronic disease. Perceived reduction in quality of life was associated with being on clinical rotation, difficulties affording healthcare services, having chronic disease, obesity, and having symptoms of COVID-19. Future studies could be done to explore further the

479 specific impact of COVID-19 on students of different major in regard to their curriculum, risk 480 and exposure in the pandemic.

481

### REFERENCES

[1] Zhu N, Zhang D, Wang W, Li X, Yang B, Song J, et al. A Novel Coronavirus from Patients with Pneumonia in China, 2019. N Engl J Med 2020;382:727–33. https://doi.org/10.1056/NEJMoa2001017.
[2] Ibn-Mohammed T, Mustapha KB, Godsell J, Adamu Z, Babatunde KA, Akintade DD, et al. A critical analysis of the impacts of COVID-19 on the global economy and ecosystems and opportunities for circular economy strategies. Resour Conserv Recycl 2021;164:105169. https://doi.org/10.1016/j.resconrec.2020.105169.
[3] Ozili PK, Arun T. Spillover of COVID-19: Impact on the Global Economy. Rochester, NY: Social Science Research Network; 2020. https://doi.org/10.2139/ssrn.3562570.
[4] Bueno-Notivol J, Gracia-García P, Olaya B, Lasheras I, López-Antón R, Santabárbara J. Prevalence of depression during the COVID-19 outbreak: A meta-analysis of community-based studies. Int J Clin Health Psychol 2021;21:100196. https://doi.org/10.1016/j.ijchp.2020.07.007.
[5] Xiong J, Lipsitz O, Nasri F, Lui LMW, Gill H, Phan L, et al. Impact of COVID-19 pandemic on mental health in the general population: A systematic review. J Affect Disord 2020;277:55–64.

https://doi.org/10.1016/j.jad.2020.08.001.

[6] Levin AT, Hanage WP, Owusu-Boaitey N, Cochran KB, Walsh SP, Meyerowitz-Katz G. Assessing the age specificity of infection fatality rates for COVID-19: systematic review, meta-analysis, and public policy implications. Eur J Epidemiol 2020;35:1123–38. https://doi.org/10.1007/s10654-020-00698-1.
[7] Nile SH, Nile A, Qiu J, Li L, Jia X, Kai G. COVID-19: Pathogenesis, cytokine storm and therapeutic potential of interferons. Cytokine Growth Factor Rev 2020;53:66–70. https://doi.org/10.1016/j.cytogfr.2020.05.002.
[8] Ali I, Alharbi OML. COVID-19: Disease, management, treatment, and social impact. Sci Total Environ 2020;728:138861. https://doi.org/10.1016/j.scitotenv.2020.138861.
[9] Pappa S, Ntella V, Giannakas T, Giannakoulis VG, Papoutsi E, Katsaounou P. Prevalence of depression, anxiety, and insomnia among healthcare workers during the COVID-19 pandemic: A systematic review and metaanalysis. Brain Behav Immun 2020;88:901–7. https://doi.org/10.1016/j.bbi.2020.05.026.
[10] Nguyen LH, Drew DA, Graham MS, Joshi AD, Guo C-G, Ma W, et al. Risk of COVID-19 among front-line healthcare workers and the general community: a prospective cohort study. Lancet Public Health 2020;5:e475–83. https://doi.org/10.1016/S2468-2667(20)30164-X.
[11] Reger MA, Piccirillo ML, Buchman-Schmitt JM. COVID-19, Mental Health, and Suicide Risk Among Health Care Workers: Looking Beyond the Crisis. J Clin Psychiatry 2020;81:0–0. https://doi.org/10.4088/JCP.20com13381.
[12] Shreffler J, Petrey J, Huecker M. The Impact of COVID-19 on Healthcare Worker Wellness: A Scoping Review.

West J Emerg Med 2020;21:1059–66. https://doi.org/10.5811/westjem.2020.7.48684.

[13] Erschens R, Keifenheim KE, Herrmann-Werner A, Loda T, Schwille-Kiuntke J, Bugaj TJ, et al. Professional burnout among medical students: Systematic literature review and meta-analysis. Med Teach 2019;41:172–83.

https://doi.org/10.1080/0142159X.2018.1457213.

[14] Tian-Ci Quek T, Wai-San Tam W, X. Tran B, Zhang M, Zhang Z, Su-Hui Ho C, et al. The Global Prevalence of Anxiety Among Medical Students: A Meta-Analysis. Int J Environ Res Public Health 2019;16:2735.

https://doi.org/10.3390/ijerph16152735.

[15] Puthran R, Zhang MWB, Tam WW, Ho RC. Prevalence of depression amongst medical students: a metaanalysis. Med Educ 2016;50:456–68. https://doi.org/10.1111/medu.12962.
[16] Meda N, Pardini S, Slongo I, Bodini L, Zordan MA, Rigobello P, et al. Students’ mental health problems before, during, and after COVID-19 lockdown in Italy. J Psychiatr Res 2021;134:69–77.

https://doi.org/10.1016/j.jpsychires.2020.12.045.

[17] Deng J, Zhou F, Hou W, Silver Z, Wong CY, Chang O, et al. The prevalence of depressive symptoms, anxiety symptoms and sleep disturbance in higher education students during the COVID-19 pandemic: A systematic review and meta-analysis. Psychiatry Res 2021:113863. https://doi.org/10.1016/j.psychres.2021.113863.
[18] Ahmed H, Allaf M, Elghazaly H. COVID-19 and medical education. Lancet Infect Dis 2020;20:777–8. https://doi.org/10.1016/S1473-3099(20)30226-7.
[19] Choi B, Jegatheeswaran L, Minocha A, Alhilani M, Nakhoul M, Mutengesa E. The impact of the COVID-19 pandemic on final year medical students in the United Kingdom: a national survey. BMC Med Educ 2020;20:206. https://doi.org/10.1186/s12909-020-02117-1.
[20] Rose S. Medical Student Education in the Time of COVID-19. JAMA 2020;323:2131–2.

https://doi.org/10.1001/jama.2020.5227.

[21] Tran BX, Vo LH, Phan HT, Pham HQ, Vu GT, Le HT, et al. Mobilizing medical students for COVID-19 responses: Experience of Vietnam. J Glob Health 2020;10:020319. https://doi.org/10.7189/jogh.10.020319.
[22] Fan AP, Tran DT, Kosik RO, Mandell GA, Hsu HS, Chen YS. Medical education in Vietnam. Med Teach 2012;34:103–7. https://doi.org/10.3109/0142159X.2011.613499.
[23] Jarrett SL, Hummel F, Whitney KL. Preparing for the 21st Century: Graduate Nursing Education in Vietnam. Nurs Educ Perspect 2005;26:172–5.
[24] Linh TNQ, Hanh TTT, Shaw R. COVID-19 initial preparedness and response in Vietnam during the first six months of the pandemic and the lessons for Sendai framework implementation. Int J Disaster Resil Built Environ 2020;12:143–55. https://doi.org/10.1108/IJDRBE-07-2020-0080.
[25] Trevisan M, Le LC, Le AV. The COVID-19 Pandemic: A View From Vietnam. Am J Public Health 2020;110:1152–3. https://doi.org/10.2105/AJPH.2020.305751.
[26] Ha BTT, Ngoc Quang L, Mirzoev T, Tai NT, Thai PQ, Dinh PC. Combating the COVID-19 Epidemic: Experiences from Vietnam. Int J Environ Res Public Health 2020;17:3125. https://doi.org/10.3390/ijerph17093125.
[27] Huynh TLD. The COVID-19 containment in Vietnam: What are we doing? J Glob Health n.d.;10.

https://doi.org/10.7189/jogh.10.010338.

[28] Xuan Tran B, Thi Nguyen H, Quang Pham H, Thi Le H, Thu Vu G, Latkin CA, et al. Capacity of local authority and community on epidemic response in Vietnam: Implication for COVID-19 preparedness. Saf Sci 2020;130:104867. https://doi.org/10.1016/j.ssci.2020.104867.
[29] Nguyen THD, Vu DC. Summary of the COVID-19 outbreak in Vietnam – Lessons and suggestions. Travel Med Infect Dis 2020;37:101651. https://doi.org/10.1016/j.tmaid.2020.101651.
[30] Hệ thống thông tin quản lý Khai báo Y tế. tokhaiyte.vn n.d. http://tokhaiyte.vn (accessed July 22, 2021).
[31] WHO Expert Consultation. Appropriate body-mass index for Asian populations and its implications for policy and intervention strategies. Lancet Lond Engl 2004;363:157–63. https://doi.org/10.1016/S01406736(03)15268-3.
[32] Nguyen HT, Do BN, Pham KM, Kim GB, Dam HTB, Nguyen TT, et al. Fear of COVID-19 Scale—Associations of Its Scores with Health Literacy and Health-Related Behaviors among Medical Students. Int J Environ Res Public Health 2020;17:4164. https://doi.org/10.3390/ijerph17114164.
[33] Ahorsu DK, Lin C-Y, Imani V, Saffari M, Griffiths MD, Pakpour AH. The Fear of COVID-19 Scale: Development and Initial Validation. Int J Ment Health Addict 2020:1–9. https://doi.org/10.1007/s11469-020-00270-8.
[34] Hawthorne G, Osborne RH, Taylor A, Sansoni J. The SF36 Version 2: Critical Analyses of Population Weights, Scoring Algorithms and Population Norms. Qual Life Res 2007;16:661–73.
[35] Ware JE, Kosinski MA, Dewey JE. How to score version 2 of the SF-36 health survey 2000.
[36] Watkins RE, Plant AJ, Sang D, O’Rourke T, Gushulak B. Development of a Vietnamese version of the Short form36 Health Survey. Asia Pac J Public Health 2000;12:118–23. https://doi.org/10.1177/101053950001200211.
[37] Burmeister E, Aitken LM. Sample size: How many is enough? Aust Crit Care 2012;25:271–4.

https://doi.org/10.1016/j.aucc.2012.07.002.

[38] Aluísio JD Barros, Vânia N Hirakata. Alternatives for logistic regression in cross-sectional studies: an empirical comparison of models that directly estimate the prevalence ratio. BMC Med Res Methodol 2003;3:1–13. [39] Zou G. A modified poisson regression approach to prospective studies with binary data. Am J Epidemiol 2004;Volume 159:702–6.
[40] Henning MA, Krägeloh CU, Hawken SJ, Zhao Y, Doherty I. The Quality of Life of Medical Students Studying in New Zealand: A Comparison With Nonmedical Students and a General Population Reference Group. Teach Learn Med 2012;24:334–40. https://doi.org/10.1080/10401334.2012.715261.
[41] Messina G, Quercioli C, Troiano G. Italian medical students quality of life: years 2005-2015. Ann Ig Med Prev E Comunità 2016:245–51. https://doi.org/10.7416/ai.2016.2103.
[42] Pham T, Bui L, Nguyen A, Nguyen B, Tran P, Vu P, et al. The prevalence of depression and associated risk factors among medical students: An untold story in Vietnam. PLOS ONE 2019;14:e0221432.

https://doi.org/10.1371/journal.pone.0221432.

[43] Majumdar P, Biswas A, Sahu S. COVID-19 pandemic and lockdown: cause of sleep disruption, depression, somatic pain, and increased screen exposure of office workers and students of India. Chronobiol Int 2020;37:1191–200. https://doi.org/10.1080/07420528.2020.1786107.
[44] Arslan G. Loneliness, college belongingness, subjective vitality, and psychological adjustment during coronavirus pandemic: Development of the College Belongingness Questionnaire. J Posit Sch Psychol 2021;5:17–31. https://doi.org/10.47602/jpsp.v5i1.240.
[45] Labrague LJ, Ballad CA. LOCKDOWN FATIGUE AMONG COLLEGE STUDENTS DURING THE COVID-19 PANDEMIC: PREDICTIVE ROLE OF PERSONAL RESILIENCE, COPING BEHAVIOURS, AND HEALTH. MedRxiv 2020:2020.10.18.20213942. https://doi.org/10.1101/2020.10.18.20213942.
[46] Vo TQ, Nguyen HTT, Ta APN. Effect of sociodemographic factors on quality of life of medical students in southern Vietnam: a survey using the WHOQOL-BREF assessment n.d.:15.
[47] Gallo LA, Gallo TF, Young SL, Moritz KM, Akison LK. The impact of isolation measures due to COVID-19 on energy intake and physical activity levels in Australian university students. MedRxiv 2020:2020.05.10.20076414. https://doi.org/10.1101/2020.05.10.20076414.
[48] Wilson OWA, Holland KE, Elliott LD, Duffey M, Bopp M. The Impact of the COVID-19 Pandemic on US College Students’ Physical Activity and Mental Health. J Phys Act Health 2021;18:272–8.

https://doi.org/10.1123/jpah.2020-0325.

[49] Luciano F, Cenacchi V, Vegro V, Pavei G. COVID-19 lockdown: Physical activity, sedentary behaviour and sleep in Italian medicine students. Eur J Sport Sci 2020;0:1–10. https://doi.org/10.1080/17461391.2020.1842910.
[50] Frajerman A, Morvan Y, Krebs M-O, Gorwood P, Chaumette B. Burnout in medical students before residency: A systematic review and meta-analysis. Eur Psychiatry 2019;55:36–42.

https://doi.org/10.1016/j.eurpsy.2018.08.006.

[51] Solis AC, Lotufo-Neto F, Solis AC, Lotufo-Neto F. Predictors of quality of life in Brazilian medical students: a systematic review and meta-analysis. Braz J Psychiatry 2019;41:556–67. https://doi.org/10.1590/1516-44462018-0116.
[52] Counsell A, Furtado M, Iorio C, Anand L, Canzonieri A, Fine A, et al. Intolerance of uncertainty, social anxiety, and generalized anxiety: Differences by diagnosis and symptoms. Psychiatry Res 2017;252:63–9.

https://doi.org/10.1016/j.psychres.2017.02.046.

# Supporting information

## S1 Table. PCS scores categories by reference population average S2 Table. MCS scores categories by reference population average S3 Fig. Residuals versus fitted values plot of PCS scores linear regression model S4 Fig. Residuals versus fitted values plot of MCS scores linear regression model S5 Table. Quantile regression analysis of PCS S6 Table. Quantile regression analysis of MCS S7 Table. Changes in quality of life before and after the pandemic S8 Table. SF-36 PCS and MCS scores with z-score derived from USA population averages

supplemental

[Click here to access/download Supporting Information](https://www.editorialmanager.com/pone/download.aspx?id=31182390&guid=4e82ad01-ef24-4019-b80a-51a023e781e3&scheme=1)

[Supplemental_20012022.docx](https://www.editorialmanager.com/pone/download.aspx?id=31182390&guid=4e82ad01-ef24-4019-b80a-51a023e781e3&scheme=1)

rebuttal letter

Dear Editors,

Sincerest thanks for the previous editor’s and reviewers’ comments on our manuscript.

We hope that a revised version of the manuscript will still be considered by PLOS One. We have modified the paper in response to the extensive and insightful reviewers’ comments. Furthermore, we have rewritten sections of the manuscript and hope that this complies with the referee’s remarks. We will respond to the comments point by point.

Our answers to reviews’ question were highlighted in yellow

PONE-D-22-11803

Pattern and perceived changes in quality of life of Vietnamese medical and nursing students during the COVID-19 pandemic

PLOS ONE

Dear Dr. Pham,

Thank you very much for submitting your manuscript for review by PLOS ONE. After careful consideration, we have decided that your manuscript does not meet our criteria and is therefore not suitable for publication in PLOS ONE.

After internal review of your manuscript, we had several concerns with the analyses, conclusions and quality of the scientific reporting. Please note that as per our publication criteria, PLOS ONE requires that all experiments, statistics and other analyses are performed to a high technical standard, described in sufficient detail and adhere to appropriate reporting guidelines and community standards. Conclusions must be presented in an appropriate fashion and be supported by the data (please see [http://journals.plos.org/plosone/s/criteria-for-publication)](http://journals.plos.org/plosone/s/criteria-for-publication). After careful consideration, we feel that the current manuscript does not meet these requirements.

For example you have not provided us with sufficient information regarding the steps taken to fully validate the additional questionnaire questions used for data collection in the study.

Thank you for your comments. We added the following information to our method section:

“The questionnaire included questions regarding *demographic information*, *academic information* and *health information*, *the Fear of Covid 19 Scale (FCV19S)*, and the *Quality of life SF-36 version 2.0 (SF-36)*, and *a question asking about the change in the general quality of life before and during the pandemic*.” “*Fear of Covid-19* was assessed using the FCV-19S questionnaire. The FCV-19S questionnaire was translated into Vietnamese in a previous study and showed good item-scale convergent validity (mean of Rho = 0.77), discriminant validity and construct validity and high internal consistency (Cronbach’s alpha = 0.90) with the Vietnamese translation [32,33].”

“The Vietnamese version of the SF-36 was validated and was shown to provide a valid assessment of self-reported health status among the Vietnamese population, with all but two of the 36 items displayed good discriminant validity and all eight scale had good discriminant validity displayed the internal consistency of the 8 scales: PF (0.82), RP (0.86), BP (0.58), GH (0.66), VT(0.56), SF (0.67), RE (0.70), MH (0.55) [36].”

As you can see, all of the main questionnaires were validated in previous studies among Vietnamese population. Furthermore, additional information in the survey were just general demographic information, academic information, and health information, such as age, gender, height, weight. Finally, the main aim of this study is not validating these questionnaires (as they were already validated in previous studies among the same population)

In addition, you have not provided sufficient details on the statistical calculations employed to determine the sample size required to power the study.

| Thank you for your comments. We added the following information to our method | |
| --- | --- |
| section: |  |

**Sample size and data collection**

The required sample size was estimated by using the formula for estimating sample mean [37], as follows:

𝑍2 𝛼∗𝜎2	2	2

𝑛 = 1−22	= 1.96 ∗212.5	= 600.25

𝑑	1

With, n being the required sample size, Z being the standard error associated with the chosen level of confidence 5%, σ being the standard deviation of the general quality of life score taken from the population used to validate the Vietnamese version of the SF36 [36].

For these reasons, we are rejecting your manuscript. I am sorry that we cannot be more positive on this occasion, but hope that you understand the reasons for this decision.

Kind regards,

Marianne Clemence

Staff Editor PLOS ONE

Additional Editor Comments (if provided):

[Note: HTML markup is below. Please do not edit.]

Reviewers' comments:

[NOTE: If reviewer comments were submitted as an attachment file, they will be attached to this email and accessible via the submission site. Please log into your account, locate the manuscript record, and check for the action link "View Attachments". If this link does not appear, there are no attachment files.]

- - - - -

For journal use only: PONEDEC3
