# Supplementary material for: Pattern and perceived changes in quality of life of Vietnamese medical and nursing students during the COVID-19 pandemic
Source: PLoS One. 2022 Dec 22;17(12):e0279446. doi: 10.1371/journal.pone.0279446 (PMC9778935; doi:10.1371/journal.pone.0279446)
Supplement: Attachment — Submitted filename: Answer to reviewers comment_TP.docx [file pone.0279446.s004.docx]

Dear Editors and Reviewers,

Sincerest thanks for the previous editor’s and reviewers’ comments on our manuscript.

We hope that a revised version of the manuscript will still be considered by PLOS One. We have modified the paper in response to the extensive and insightful reviewers’ comments.

Furthermore, we have rewritten sections of the manuscript and hope that this complies with the reviewers’ remarks. We will respond to the comments point by point.

**All the line number mentioned in this document were based on the marked-up copy (the revised manuscript with track changes).**

PONE-D-22-14424

Pattern and perceived changes in quality of life of Vietnamese medical and nursing students during the COVID-19 pandemic

PLOS ONE

Dear Dr. Pham,

Thank you for submitting your manuscript to PLOS ONE. After careful consideration, we feel that it has merit but does not fully meet PLOS ONE’s publication criteria as it currently stands. Therefore, we invite you to submit a revised version of the manuscript that addresses the points raised during the review process.

Three reviewers have engaged with your study. All the reviewers see your study relevant and with the potential for publication subject to some revisions. Please critically engage with the reviewer comments and resubmit for reconsideration.

Please submit your revised manuscript by Aug 22 2022 11:59PM. If you will need more time than this to complete your revisions, please reply to this message or contact the journal office at [plosone@plos.org](mailto:plosone@plos.org). When you're ready to submit your revision, log on to <https://www.editorialmanager.com/pone/> and select the 'Submissions Needing Revision' folder to locate your manuscript file.

Please include the following items when submitting your revised manuscript:

- A rebuttal letter that responds to each point raised by the academic editor and reviewer(s). You should upload this letter as a separate file labeled 'Response to Reviewers'.
- A marked-up copy of your manuscript that highlights changes made to the original version. You should upload this as a separate file labeled 'Revised Manuscript with Track Changes'.
- An unmarked version of your revised paper without tracked changes. You should upload this as a separate file labeled 'Manuscript'.

If you would like to make changes to your financial disclosure, please include your updated statement in your cover letter. Guidelines for resubmitting your figure files are available below the reviewer comments at the end of this letter.

If applicable, we recommend that you deposit your laboratory protocols in [protocols.io](http://protocols.io/) to enhance the reproducibility of your results. Protocols.io assigns your protocol its own identifier (DOI) so that it can be cited independently in the future. For instructions see: <https://journals.plos.org/plosone/s/submission-guidelines#loc-laboratory-protocols>. Additionally, PLOS ONE offers an option for publishing peer-reviewed Lab Protocol articles, which describe protocols hosted on [protocols.io](http://protocols.io/). Read more information on sharing protocols at <https://plos.org/protocols?utm_medium=editorial-email&utm_source=authorletters&utm_campaign=protocols>.

We look forward to receiving your revised manuscript.

Kind regards,

Christmal Dela Christmals, PhD, MSc, BSc, RN
Academic Editor
PLOS ONE

Journal Requirements:

When submitting your revision, we need you to address these additional requirements.

1. Please ensure that your manuscript meets PLOS ONE's style requirements, including those for file naming. The PLOS ONE style templates can be found at 
<https://journals.plos.org/plosone/s/file?id=wjVg/PLOSOne_formatting_sample_main_body.pdf> and 
<https://journals.plos.org/plosone/s/file?id=ba62/PLOSOne_formatting_sample_title_authors_affiliations.pdf>

Thank you for your comments, we checked and revised the manuscript to meet the PLOS ONE's style requirements.

2. Please provide additional details regarding participant consent. In the ethics statement in the Methods and online submission information, please ensure that you have specified what type you obtained (for instance, written or verbal, and if verbal, how it was documented and witnessed). If your study included minors, state whether you obtained consent from parents or guardians. If the need for consent was waived by the ethics committee, please include this information.

Once you have amended this/these statement(s) in the Methods section of the manuscript, please add the same text to the “Ethics Statement” field of the submission form (via “Edit Submission”).

Thank you for your comments, all participants received comprehensive information about the survey and so were fully informed before giving consent (by clicking “I agree to participate” button on the informed consent page of the questionnaire). We added this information on the “Ethical issue” section as you requested (Line 251)

For additional information about PLOS ONE ethical requirements for human subjects research, please refer to <http://journals.plos.org/plosone/s/submission-guidelines#loc-human-subjects-research>.

3. We note that you have indicated that data from this study are available upon request. PLOS only allows data to be available upon request if there are legal or ethical restrictions on sharing data publicly. For more information on unacceptable data access restrictions, please see <http://journals.plos.org/plosone/s/data-availability#loc-unacceptable-data-access-restrictions>.

In your revised cover letter, please address the following prompts:

a) If there are ethical or legal restrictions on sharing a de-identified data set, please explain them in detail (e.g., data contain potentially sensitive information, data are owned by a third-party organization, etc.) and who has imposed them (e.g., an ethics committee). Please also provide contact information for a data access committee, ethics committee, or other institutional body to which data requests may be sent.

b) If there are no restrictions, please upload the minimal anonymized data set necessary to replicate your study findings as either Supporting Information files or to a stable, public repository and provide us with the relevant URLs, DOIs, or accession numbers. For a list of acceptable repositories, please see <http://journals.plos.org/plosone/s/data-availability#loc-recommended-repositories>.

We will update your Data Availability statement on your behalf to reflect the information you provide.

According to our application to the Institutional Review Board (IRB) of Hanoi University of Public Health, the data cannot be shared publicly because of ethical restrictions to protect the confidentiality of the participants imposed by the IRB. A de-identified dataset is available for researchers who meet the criteria for access to confidential data. Requests for data should be submitted to Institutional Review Board of Hanoi University of Public Health

(irb@huph.edu.vn) and the corresponding author, Dr. Pham Thanh Tung ([phamthanhtung@hmu.edu.vn](mailto:phamthanhtung@hmu.edu.vn))

4. We note you have included a table to which you do not refer in the text of your manuscript. Please ensure that you refer to Table 3 an 4 in your text; if accepted, production will need this reference to link the reader to the Table.

[Note: HTML markup is below. Please do not edit.]

Reviewers' comments:

Reviewer's Responses to Questions

**Comments to the Author**

**Reviewer 1 Comments**

Thank you for your very insightful comments. These comments greatly helped us complete and improve our manuscript. We will be replying to every specific comment down below.

**Comment:**

The manuscript is technically sound and the data support all the conclusions made
L26- L 61 Credentials – some authors do not have Orcid numbers. It will be beneficial to generate an Orcid number
L 64 ABSTRACT- abstract is concise and succinct.

**Answer:** Thank you for your comments. We will ask all of our co-authors to include this information

**Comment:** L67 add nursing to medical students

**Answer:** we have fixed the sentence as recommended in the manuscript

**Comment:** L 101 add s to student

**Answer:** we have added “s” to student in the keyword section as recommended

**Comment:**

Introduction – well explained and purpose of the study well explained
Methods
The test and sample size are appropriate
The language used is clear, correct, and unambiguous
Setting – well explained
Survey instrument – questionnaire items well explained

**Answer:** Thank you for your comments

Sample size
**Comment:** L221 - remove full stop after percentage of nursing students

**Answer:** Thank you for your comments. We have removed the full stop in the sentence

**Comment:** L223 – Replace “an” with a in “A self-reported…”

**Answer:** Thank you for your comments. We have replaced the “an” with “a” in the sentence.

**Comment:** L225 – Who are these representatives?

**Answer:** Thank you for your comments. We added the following information on line 228

“The class representatives are students that are responsible for major communication between the school administration and the student of the class that they’re in. There is one class representative for each class, and this practice is common for most universities in Vietnam”

Data analysis
**Comment:** L235 – Separate the ap-value to read a p-value. Data analysis procedure explained well

**Answer:** Thank you for your comments. we have fixed the sentence as recommended.

**Comment:**

Ethical issues are well explained by authors
Results
Results well explained
Table concise

**Answer:** Thank you for your comments

**Comment:** L291 – What does normal students mean? Try another word to describe them as this does not sound good

**Answer:** Thank you for your comments. We have rephrased the sentence as below on line 297

“The PCS score was significantly lower in those who had symptoms of COVID-19 (p <0.001) and those who have significant fear of COVID-19 (p <0.001), compared to those who didn’t.”

**Comment:**
Discussion
Discussion covered the main findings in the study and exposed challenges face by health students on clinical rotation during the Covid -19 pandemic.
Strengths and limitations
Strengths and limitations well explained in the study
**Answer:** Thank you for your comments

Conclusion
**Comment**: Replace sample on L471 with group names of participants

**Answer:** Thank you for your comments. We have rephrased the sentence as below on line 477

“The physical and mental quality of life of the medical and nursing students was lower than that of the general population”

**Reviewer 2 Comments**

Thank you for your very insightful comments. These comments greatly helped us complete and improve our manuscript. We will be replying to every specific comment down below.

**Comment:** How many is all?

**Answer:** Thank you for your comments. The total number of participants was 3672 students. We revised line 75-76 as follows:

“The study was a cross-sectional study on all students of Hanoi Medical University from 3 majors: General Medicine, Preventive Medicine, Nursing (3672 invited students)”

**Comment:** The response rate seems low

**Answer:** Thank you for your comments. Due to using online questionnaires to collect data, the response rate is usually low. This was acknowledged in our limitation section in the full text on line 459:

“However, this study used an online, anonymous data collection scheme, which lead to a low response rate of only about 43%, and potential sampling errors, and selection bias.”

**Comment:** Would be good to highlight, what leads to the previous mental problem faced by the students for the reader to follow the progression of the risk factors.

**Answer:** Thank you for your comments. We revised it as follows on line 116

“Medical and nursing students, before the pandemic, were already known to face multiple physical and mental problems including burnout, anxiety, depression, and other mental health issues, with stress, lack of academic motivation, and financial hardship being important risk factors [13–16]”

**Comment:** Please include literature, that address what in quality life in a life of Medical and Nursing student, and how it affects them so as to link the following sentence that address the solution which is the study aim.

**Answer:** Thank you for your comments. This sentence was intended as our hypothesis rather than information from the literature. We revised it as follows on line 126

“Together with other socio-economic aspects related to the pandemic like lockdown, social distancing, limitation in physical activities, the authors hypothesized that the physical and mental quality of life of medical students could be at high risk and can potentially lead to poorer health outcomes.”

**Comment:** The authors, instead of we.

**Answer:** Thank you for your comments. We revised the sentence as you suggested

**Comment:** Would be good to add the overall medical and nursing students that were enrolled in that period for the reader to see the sample size you got for the study.
**Answer:** Thank you for your comments. We revised the sentence as you suggested on line 138.

“A cross-sectional study was conducted on 1583 students who major in medicine, preventive medicine, and nursing, at Hanoi Medical University (out of total 3672 students), from 7th to 29th of April 2020. This period is within the first 6 months of the covid-19 pandemic in Vietnam”

**Comment:** Ensure consistency. Second or 2nd ?

**Answer:** Thank you for your comments. We have fixed the typing error (from 2^nd^ to second) to ensure consistency

**Comment:** Is the instrument developed, adapted or edited by the authors and is the permission ranted if it was adapted ?

**Answer:** Thank you for your comments. We included detailed information on these questionnaires in our method section. No adaptation or edition was made by the authors as we simply used the Vietnamese version (adapted, translated, tested, and sometimes validated in previous studies)

Line 176 “The FCV-19S questionnaire was translated into Vietnamese in a previous study and showed good item-scale convergent validity (mean of Rho = 0.77), discriminant validity and construct validity and high internal consistency (Cronbach’s alpha = 0.90) with the Vietnamese translation [33,34]”. The information on this questionnaire was available online from previous paper and could be considered resource on public domain, which could be reused by other researchers.

Line 187 “The quality of life of students during the period was determined using the Vietnamese version of the Quality-of-life SF-36 version 2.0 [35]”. The information on this questionnaire was available online from previous paper and could be considered resource on public domain, which could be reused by other researchers.

The license for this questionnaire was also fully granted by RAND: https://www.rand.org/health-care/surveys_tools/mos/36-item-short-form/terms.html

**Comment:** Acknowledge the source, since it is adapted please

**Answer:** Thank you for your comments. We included the name of the author of the article in the source and cited accordingly on line 177

“Fear of Covid-19 was assessed using the FCV-19S questionnaire. The FCV-19S questionnaire was translated into Vietnamese in a previous study by Nguyen and colleague and showed good item-scale convergent validity (mean of Rho = 0.77), discriminant validity and construct validity and high internal consistency (Cronbach’s alpha = 0.90) with the Vietnamese translation [33,34].”

**Comment:** Suggested a usage of nor instead of or

**Answer:** Thank you for your comments. We have changed it to “neutral” as in the original English version

https://www.nlm.nih.gov/dr2/Fear_of_Covid-19_Scale_2020.pdf

Line 180: “It consisted of 7 items and utilized a 5-point Likert scale with 1 = “strongly disagree”, 2 = “disagree”, 3 = “neutral”, 4 = “agree”, 5 = “strongly agree””

**Comment:** What informed then number of invited participants. It needs be scientifically justifiable

**Answer:** Thank you for your comments. The total number of invited students was collected using record from the administration. As for the scientifical justification, we invited ALL students in Doctor of General Medicine, Doctor of Preventive Medicine, and Bachelor of Nursing tracks (3672 students) rather than using random sampling. Inviting all individuals from the target population to participate is always the best sampling method if resources are available.

We also provided information on sample size calculations on line 211:

**“Sample size and data collection**

The required sample size was estimated by using the formula for estimating sample mean [38], as follows:

$n=\frac{Z_{1-\frac{\alpha}{2}}^{2}{*\sigma}^{2}}{d^{2}}=\frac{{1.96}^{2}*{12.5}^{2}}{1^{2}}=600.25$

With, n being the required sample size, Z being the standard error associated with the chosen level of confidence 5%, σ being the standard deviation of the general quality of life score taken from the population used to validate the Vietnamese version of the SF-36 [35].”

**Comment:** Clarify the ap to the reader

**Answer:** Thank you for your comments. It is a typo, we changed it to:

Line 241: “We consider a p -value < 0.05 as statistically significant for all statistical tests”

**Comment:** Add a source to reference this, it is significant but elaborate as to according to who

**Answer:** Thank you for your comments. We included citation on paper tracking and discussing the origin of this threshold. We understand this threshold is not perfect; therefore, we always try to provide full p-value rather than using cut-off and also use 95%CI when possible.

**Comment:** Provide value to the reader

**Answer:** Thank you for your comments. We used general medicine was used as a reference in the regression model for the independent variable of major, so the coefficient for general medicine was 0 while the coefficient for nursing was not significantly different from the reference, but the table did include this information. The coefficient could be understood as the difference among groups when comparing the score.

We also added detailed values in the following sentence on line 337:

“Regarding academic characteristics, majoring in Preventive Medicine result in higher MCS score (Coef: 2.97; 95% CI: 0.33 to 5.6) compared to those who were majoring in general medicine. Lower MCS score was associated with: having chronic disease (Coef: -9.5; 95% CI: -1.7 to -5.6), having atypical symptoms of COVID-19 (Coef: -7.59; 95% CI: -12 to -3.4), difficulties affording healthcare services (Coef: -5.20; 95% CI: -7.2 to -3.2). The association of having atypical symptoms and affordability of healthcare services with MCS score was also consistent with our quantile regression analysis. However, in the quantile regression analysis with the same variables (S6 Table), there were no association between the Preventive Medicine major (p=0.064), chronic disease and MCS (p=0.073), but being on clinical rotation was associated with a higher MCS score (Coef: 3.239; 95% CI: 0.71 to 5.8).”

**Comment:** Delete douplicate

**Answer:** Thank you for your comments we deleted the duplicate

**Comment:** This affirms the earlier suggestion, that if the tool is amended. Its reliability and validity must be ensured in the current context

**Answer:** Thank you for your comments. All questionnaires in languages other than English usually involve translation. Although these questionnaires may be validated rigorously. The reliability and validity will never be 100% as the original version in the original language. Even a sensitivity and specificity of 99% is not 100%. Therefore, in this sentence, we want to clearly state this fact even though previous studies may have already validated these tools rigorously in Vietnamese population.

**Reviewer 3 Comments**

Thank you for your very insightful comments. These comments greatly helped us complete and improve our manuscript. We will be replying to every specific comment down below.

**Comment:** The experiments and the statistical analysis seem to have been conducted rigorously. Data underlying the findings is not made fully available. The authors in the manuscript indicated the following in this regards: "According to our application to I have no competing interests, the data cannot be shared publicly because of ethical restrictions to protect the confidentiality of the participants. A de-identified dataset is available for researchers who meet the criteria for access to confidential data." The reviewer understands and respect that the Institutional Review Board of Hanoi University of Public Health wants to protect the confidentiality of participants in this way. The reviewer in this regard wants to suggest that the authors should consider omitting the name of the University to not only further protect the confidentiality of the participants but also of the University.

**Answer:** Thank you for your comments. The dataset of this paper was collected at only one university, and the IRB did not prohibit us from reporting the name of the university from this data as there is no sensitive data regarding the university. We think that reporting group level data like table and figure are good enough to protect the confidentiality of participants

**Comment:** In rule 136-138 the authors mentioned that the cross-sectional study was conducted in the first six months of the COVID-19 pandemic and then go further to state the dates on which the study was conducted namely 7-29 April of 2020. The sentence could cause confusion as it could sound as if the study was conducted over a period of six months.

**Answer:** Thank you for your comments. We have adjusted the sentence in the manuscript as follows, in order to clear the confusion.

Line 138: “A cross-sectional study was conducted on 1583 students major in medicine, preventive medicine, and nursing, at Hanoi Medical University (out of total 3672 students), from 7th to 29th of April 2020. This period is within the first 6 months of the covid-19 pandemic in Vietnam.”

**Comment:** In the discussion of the limitations of the study, the authors seem to contradict themselves with regards to utilizing a translated version of the SF-36 and of the FCV-19S. The authors first mentioned that the main instrument of the study namely the Vietnamese version of the SF-36 was validated and as a result provided a valid assessment of self-reported health status among the Vietnamese population. It further allowed for both compound and specific evaluation of quality life. Then in the discussion of the limitation of the study, the authors indicated that the translation of the SF-36 and the FCV-19S into Vietnamese might have affected the validity of these questionnaires.

The reviewer wonders whether the research was not conducted too hastily which resulted in preventing the authors to conduct a pilot study beforehand in order to rule out the possible limitations that the translation of the documents in the end brought about.

**Answer:** Thank you for your comments. All questionnaires in languages other than English usually involve translation. Although these questionnaires may be validated rigorously. The reliability and validity will never be 100% as the original version in the original language. Even a sensitivity and a specificity of 99% is not 100%.

Therefore, in this sentence, we want to clearly state this limitation even though previous studies may have already validated these tools rigorously in Vietnamese population.

**Comment:** The reviewer wants to suggest that if the manuscript is to be published that the authors add information pertaining to what was put into place to support participants who, as a result of the study, needed psychological treatment/assistance. From the discussion of the results, it is for instance mentioned that the quality of life for some of the participants changed as a result of the COVID-19 pandemic. By participating in the study could have created a specific awareness amongst some of the participants about these changes which potentially could for instance have contributed to further stress and anxious feelings. The authors in Rules 374-375 for instance refer to what the mental state of medical students could be as a result of the sudden changes in the context of the pandemic.

**Answer:** Thank you for your comments. The questionnaire was anonymous, so we could not refer student to a clinician. This anonymous procedure was put in place to protect the participants’ identity and encourage them to participate in the study as many students with mental health issue don’t want to reveal this information. The IRB also agreed and approved this anonymous procedure.

**Comment:** The reviewer is of the opinion that the results of the specific topic lends itself to causation interpretation. The authors stated that the use of a cross-sectional study design however limited their interpretation of the results to association, rather than causation. The interpretation of the conclusions therefore seems to be very linearly done. The authors do not draw adequate connections between the outcome of the study and the quality of life of the different groups that participated.

**Answer:** Thank you for your comments. The connection between quality of life and associated factors was discussed with regards to the data collected. There are many factors that need to be considered to use causation interpretation in this case. We have a low response rate, many unmeasured/residual confounders, and weak temporarily relationship in this study. All of these factors could completely change the results: student with low quality of life don’t want to participate, a strong unmeasured confounder like academic motivation distorted the relationship, the relationship is not causal as low quality of life lead to the risk factor (not the other way).

Therefore, we are not confidence to put forward a causal interpretation and would only report association.

**Comment:** The formulation and construction of sentence for instance need attention (Refer to Rules 196 and 197: Should the formulation of the last part of the sentence not read: and any score below 50 was considered to be "Below the population average". etc).

**Answer:** Thank you for your comments. We have rephrased the sentence accordingly.

Line 198: “A PCS/MCS score of 50 represents the reference population average, and any score below 50 would be considered “Below the population average””

**Comment:** In certain sentences words such as ‘the’ and ‘a’ are missing (Refer to Rule 171: The word "the" should be added between the words "to" and WHO"s etc).

**Answer:** Thank you for your comments. We have rephrased the sentence accordingly.

Line 174: “BMI was classified into 4 categories according to the WHO Asian – Pacific cutoff point: underweight (<18.5 kg/m2), normal weight (18.5–22.9 kg/m2), overweight (23–24.9 kg/m2), and obese (≥25 kg/m2) [32]”

**Comment:** COVID-19 is sometimes indicated as Covid-19 (Refer to Rules 101, 137, 156, 174 etc).

**Answer:** Thank you for your comments. We have adjusted the sentences according to the recommendation to ensure consistency.

**Comment:** Other examples where attention needs to be paid to the editing of the manuscript involves the following: Rule 3: it should be students and not just "student" as it currently stands; Rule 136: the "who" needs to be added between the words "student" and "major".
It is strongly suggested that the manuscript should be language edited if it is to be reviewed again.

**Answer:** Thank you for your comments. We have rewritten sections of the manuscript and hope that this complies with the reviewer’ remarks
