# Supplementary material for: Pattern and perceived changes in quality of life of Vietnamese medical and nursing students during the COVID-19 pandemic
Source: PLoS One. 2022 Dec 22;17(12):e0279446. doi: 10.1371/journal.pone.0279446 (PMC9778935; doi:10.1371/journal.pone.0279446)
Supplement: Attachment — Submitted filename: Answer to reviewers comment_TP.docx [file pone.0279446.s005.docx]

Dear Editors and Reviewers,

Sincerest thanks for the previous editor’s and reviewers’ comments on our manuscript.

We hope that a revised version of the manuscript will still be considered by PLOS One. We have modified the paper in response to the extensive and insightful reviewers’ comments.

Furthermore, we have rewritten sections of the manuscript and hope that this complies with the reviewers’ remarks. We will respond to the comments point by point.

**All the line number mentioned in this document were based on the marked-up copy (the revised manuscript with track changes).**

Review Comments to the Author

Please use the space provided to explain your answers to the questions above. You may also include additional comments for the author, including concerns about dual publication, research ethics, or publication ethics. (Please upload your review as an attachment if it exceeds 20,000 characters)

Reviewer #1: REVIEW COMMENTS
Pattern and perceived changes in quality of life of Vietnamese medical and nursing students during the COVID-19 pandemic
SUMMARY OF RESEARCH AND IMPRESSION
This study looked at the patterns and perceptions of changes in quality of care of medical and nursing students during Covid -19 pandemic. This study is really timely to identify issues that students face and how institutions can support them during training. This can also serve as a guide for replication of this study in other contexts. I recommend that the manuscript be accepted and published.

Thank you for your comments!

ABSTRACT
Abstract is well explained, and major findings highlighted

Thank you for your comments!

INTRODUCTION
Introduction has more information on medical students than nursing students although the study covers both specialties. I suggest you consider more informing pertaining to nurses.

Thank you for your comments! We added several references according to your suggestion

“Healthcare workers were shown to have a significantly higher risk of COVID-19 infection, compared to the general public, and faced with extraordinary amounts of pressures leading to physical and mental exhaustion [9–14]” => We added 2 studies that look specifically at nurses in the front-line of COVID-19

“Medical and nursing students, before the pandemic, were already known to face multiple physical and mental problems including burnout, anxiety, depression, and other mental health issues, with stress, lack of academic motivation, and financial hardship being important risk factors [15–21]” => We added 2 studies that look specifically at nursing students

“Medical education also changed rapidly in response to the situation, with the replacement of in-person classes with online equivalents and disruption of clinical rotations [24–29]” => We added 2 studies that look specifically at nursing students and how their education was affected by COVID-19

METHODS
L140 – Check the sentence on this line use “was” instead of “is”

Thank you for your comments! We changed it to “is”

L152 – delete being from the sentence

Thank you for your comments! We deleted the word “being”

Data collection instruments well explained

Thank you for your comments!

L211 – not having no change – correct the two negative words

Thank you for your comments! We changed it to “having no change or improvement in their quality of life”

L222 – can you provide a brief explanation the low response rate among the general Medicine students?

Thank you for your comments! We added the explanation for this in line 462 in the limitation section

“The difference in response rate between academic majors could be due to clinical rotation’s scheduling, the willingness and interest of the class representatives, or miscommunications between the Office for Student Services and the class representatives. Due to the anonymous feature of the survey, we were not able to pinpoint the exact reasons for the difference in response rate and will look into this issue in future studies”

Statistical analysis well explained

Thank you for your comments!

RESULTS
Results well explained with required statistical methods
Tables are well labelled

Thank you for your comments!

DISCUSSION
Discussion covered the main findings of the study

Thank you for your comments!

Reviewer #3: Thank you for attending to the previous comments and recommendations that were made. There are however still a few places where COVID-19 is still referred to as Covid-19 or even covid-19. Refer to Lines 134; 140; 152 and 159.

Thank you for your comments! We did a search for all inconsistencies in wording of COVID-19 and changed it as you suggested

Line 233 starts with the word "Thorough" and it should be "Through".

Thank you for your comments! We changed it to "Through" as you pointed out

In Line 320 is should be Table 3 and not table 3. The same goes for Line 337 where it should be Table 4 and not table 4. The reason for the last two comments is because the Table elsewhere is written with a capital letter.

Thank you for your comments! We changed it to Table 3 and Table 4 as you pointed out
